# Ubiquitination of SARS-CoV-2 NSP6 and ORF7a Facilitates NF-κB Activation

**DOI:** 10.1128/mbio.00971-22

**Published:** 2022-07-20

**Authors:** Hironori Nishitsuji, Satoko Iwahori, Mariko Ohmori, Kunitada Shimotohno, Takayuki Murata

**Affiliations:** a Department of Virology and Parasitology, Fujita Health University School of Medicine, Aichi, Japan; b The Research Center for Hepatitis and Immunology, National Center for Global Health and Medicine, Chiba, Japan; Ohio State University; University of Pennsylvania

**Keywords:** inflammation, NF-κB, SARS-CoV-2

## Abstract

Patients with severe coronavirus disease 2019 tend to have high levels of proinflammatory cytokines, which eventually lead to cytokine storm and the development of acute respiratory distress syndrome. However, the detailed molecular mechanisms of proinflammatory cytokine production remain unknown. Here, we screened severe acute respiratory syndrome coronavirus 2 (SARS-CoV-2) genes and found that nonstructural protein 6 (NSP6) and open reading frame 7a (ORF7a) activated the NF-κB pathway. NSP6 and ORF7a interacted with transforming growth factor β-activated kinase 1 (TAK1), and knockout (KO) of TAK1 or NF-κB essential modulator (NEMO) abolished NF-κB activation by NSP6 and ORF7a. Interestingly, K61 of NSP6 was conjugated to K63-linked polyubiquitin chains by the E3 ubiquitin ligase tripartite motif-containing 13, and this polyubiquitination of NSP6 appeared crucial for recruitment of NEMO to the NSP6-TAK1 complex and NF-κB activation. On the other hand, ring finger protein 121 (RNF121) was required for the polyubiquitination of ORF7a. Knockdown of RNF121 significantly decreased ORF7a binding of TAK1 and NEMO, resulting in the suppression of NF-κB activation. Taken together, our results provide novel molecular insights into the pathogenesis of SARS-CoV-2 and the host immune response to SARS-CoV-2 infection.

## INTRODUCTION

Severe acute respiratory syndrome coronavirus 2 (SARS-CoV-2), the causative agent of coronavirus disease 2019 (COVID-19), emerged in 2019 and triggered a global pandemic (1). The symptoms are relatively mild during the first week after infection, but the infection later results in atypical pneumonia and acute respiratory distress syndrome in a proportion of infected individuals. Patients with severe symptoms frequently exhibit excessive production of proinflammatory cytokines, which leads to cytokine storm syndrome (2–9). The levels of proinflammatory cytokines/chemokines such as interleukin 6 (IL-6), IL-8, interferon gamma (IFN-γ)-induced protein 10 (IP-10), and tumor necrosis factor alpha (TNF-α) are markedly elevated in the sera of severe COVID-19 patients (4–9).

The robust induction of cytokines/chemokines by SARS-CoV-2 infection is not surprising. SARS-CoV-2 RNA is recognized by retinoic acid-inducible gene I (RIG-I) and melanoma differentiation-associated protein 5 (MDA5) (10), which activate downstream signaling molecules such as mitochondrial antiviral signaling protein (MAVS), TANK-binding kinase 1 (TBK1), and IFN regulatory factor 3 (IRF3), and then induce type I IFN production (11). Secreted type I IFN binds to its receptor on the cell surface and triggers the stimulation of the Janus kinase (JAK)/signal transducer and activator of transcription (STAT) signaling pathways, resulting in the expression of IFN-stimulated genes (ISGs) (12). However, previous detailed analyses suggest that cytokines/chemokines responses to SARS-CoV-2 infection are biased (9). In fact, COVID-19 patients show impaired host antiviral responses, including type I/III IFN production and the expression of ISGs. This imbalanced cytokine/chemokine response in COVID-19 patients is mediated by the attenuation of host-specific antiviral signals by SARS-CoV-2 gene products (13–16). For example, the nuclear import of STAT is blocked by open reading frame 6 (ORF6) of SARS-CoV-2 (13). ISG-15-dependent activation of MDA5 is repressed by a protease of the virus (16). Despite the suppression of host IFN signaling by SARS-CoV-2 infection, levels of cytokines/chemokines, such as IL-6, IL-8, IP-10, and TNF-α, are significantly elevated in COVID-19 patients. This can be explained partly by phosphoinositide 3-kinase, AKT, and mammalian target of rapamycin signaling activation by the spike (S) protein (17). Viral nucleocapsid (N) and envelope (E) proteins activate nucleotide-binding oligomerization domain-like receptor family pyrin domain containing 3 (NLRP3)-dependent inflammasome and Toll-like receptor 2 (TLR2) pathways, respectively, to induce those proinflammatory cytokines through activation of the NF-κB pathway (18, 19). Indeed, NF-κB is one of the most important transcription factors in the proinflammatory response to SARS-CoV-2 infection (20, 21).

The NF-κB family is composed of five members, namely, p50, p52, p65 (RelA), RelB, and c-Rel, and there are two distinct NF-κB pathways, canonical and noncanonical (22). In response to the ligation of stimulating agents, including cytokines, bacteria, and viral RNA/DNA, specific receptors recruit adaptor molecules, including TNF receptor-associated factors 2, 5, and 6 (TRAF2/TRAF5/TRAF6), (23–25) receptor-interacting proteins 1 and 2 (RIP1 and RIP2) (26, 27), and myeloid differentiation primary response gene 88 (Myd88) (28), and then activate transforming growth factor β-activated kinase 1 (TAK1) and the IκB kinase (IKK) complex (29) to trigger the canonical NF-κB pathway. Activation of these molecules allows the proteasomal degradation of IκBα after its phosphorylation and then results in p65/p50 heterodimer formation, followed by the production of proinflammatory cytokines and chemokines (30). The noncanonical pathway is initiated by the stimulation of TNF receptor (TNFR) superfamily members, including lymphotoxin β receptor, BAFF receptor (also known as TNFR superfamily member 13C), cluster of differentiation 40 (CD40), and RANK (also known as TNFR superfamily member 11A) (31–34). This pathway is mediated by NF-κB-inducing kinase (NIK) and IKKα activation (35), leading to translocation of the RelB/p52 heterodimer to the nucleus to regulate B cell survival and dendritic cell (DC) activation. Single-cell analyses have clearly demonstrated that SARS-CoV-2 infection selectively activates NF-κB signaling (21). More recent studies have shown that viral N protein promotes activation of the NF-κB pathway through interactions with TAK1 and IKK complexes (36), and the ORF7a protein induces NF-κB-dependent cytokine production (37). Importantly, inhibition of canonical NF-κB signaling reduces the cytokine storm in COVID-19 patients (38–40). In addition, silencing of the canonical NF-κB transcription factor complex (p50 or p65) using small interfering RNA (siRNA) suppresses SARS-CoV-2 replication (21). These reports indicate that NF-κB signaling is a potential therapeutic target for COVID-19. However, the involvement of other viral genes besides S, N, and ORF7a is still unknown, and the molecular mechanisms underlying NF-κB signal activation by ORF7a remain unclear.

In this study, we screened 22 SARS-CoV-2 proteins for their ability to activate NF-κB signaling, and we identified nonstructural protein 6 (NSP6) and ORF7a. The expression of NSP6 or ORF7a induced NF-κB activation and increased IL-8 and IP-10 mRNA expression in a TAK1- and NF-κB essential modulator (NEMO)-dependent manner. In addition, both NSP6 and ORF7a interacted with TAK1 and were ubiquitinated by the E3 ubiquitin ligase tripartite motif-containing 13 (TRIM13) and RNF121, respectively. Ubiquitinated NSP6 and ORF7a recruited NEMO, thus activating the NF-κB pathway. Our results demonstrate that SARS-CoV-2 NSP6 and ORF7a promote the induction of proinflammatory responses.

## RESULTS

### SARS-CoV-2 NSP6 and ORF7a activate the NF-κB pathway.

**FIG 1:**
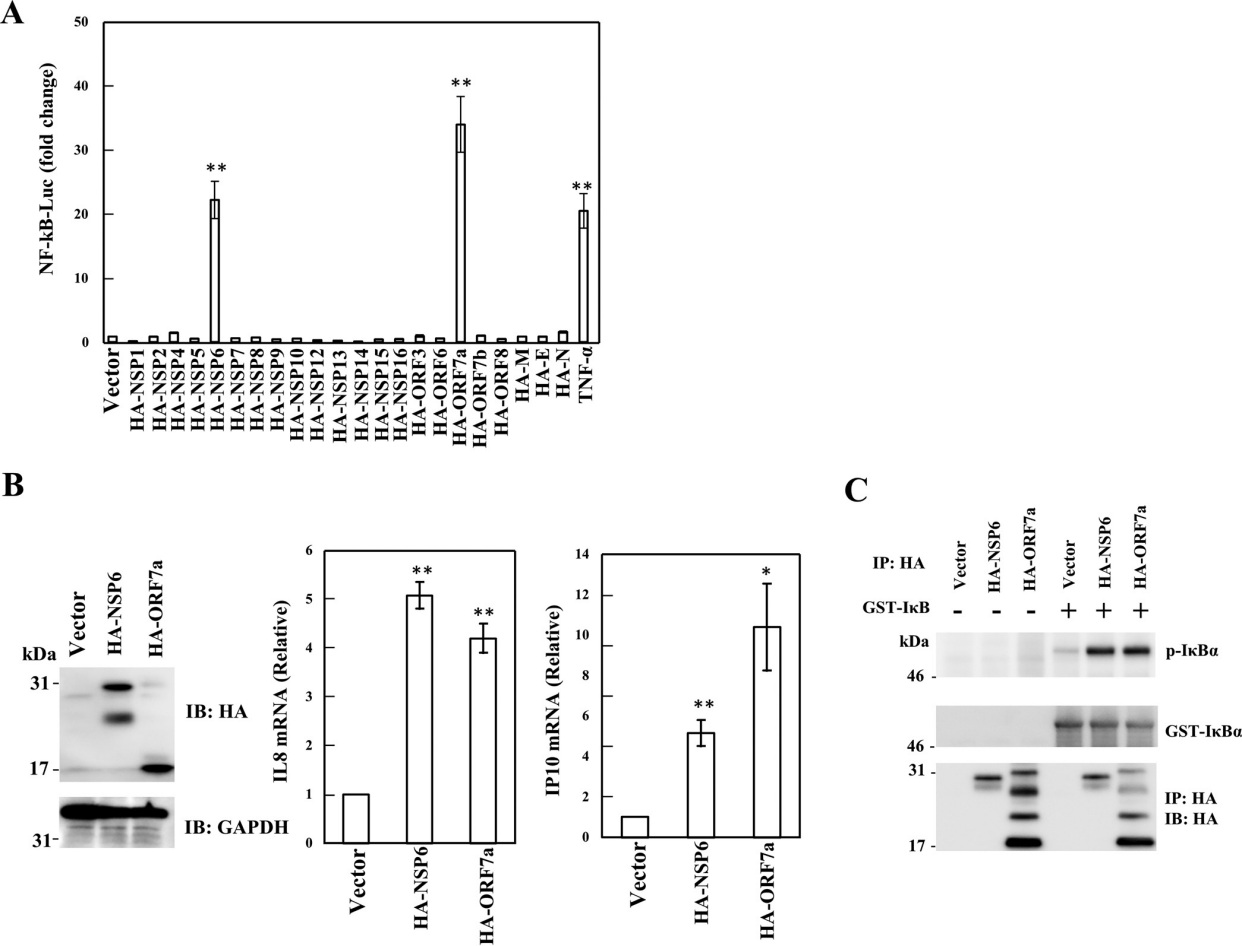
NSP6 and ORF7a induced NF-κB activation. (A) HEK293T cells were transfected with each SARS-CoV-2 protein expression plasmid, along with pGL4-NF-κB-LucP2 and pNull-RLuc. The level of luciferase activity was determined at 24 h posttransfection. The firefly luciferase activity was normalized to *Renilla* luciferase activity. An empty plasmid was used as a control, and the value was set as 1. Treatment with TNF-α (5 ng/mL) was performed for 24 h as a positive control. Results are shown as the mean ± standard deviation (SD) of three independent experiments. Statistics were generated by comparison with empty plasmid. **, *P* < 0.01 (Student’s *t* test). (B) HEK293T cells were transfected with pcDNA3.1-HA, pcDNA3.1-HA-NSP6, or pcDNA3.1-HA-ORF7a. At 48 h after transfection, the mRNA and protein levels were determined by RT-qPCR and Western blotting, respectively. The indicated mRNA levels were normalized to GAPDH expression. An empty plasmid was used as a control and set to 1. Results are shown as the mean ± SD of three independent experiments. Statistics were generated by comparison with empty plasmid. *, *P* < 0.05; **, *P* < 0.01 (Student’s *t* test). (C) For the *in vitro* kinase assay, HEK293T cells were transfected with pcDNA3.1-HA, pcDNA3.1-HA-NSP6, or pcDNA3.1-HA-ORF7a. At 48 h after transfection, cells were lysed with 1× IP lysis buffer, followed by IP with the anti-HA antibody. The immunocomplex was mixed with GST-IκBα and ATP-γ-S in the kinase buffer. Phosphorylation of GST-IκB was detected by anti-thiophosphate ester antibody.

To identify SARS-CoV-2 proteins that can regulate viral host responses, we cloned the following ORFs of the SARS-CoV-2 genome fused to an N-terminal hemagglutinin (HA) tag into a mammalian expression plasmid: NSP1, NSP2, NSP4 to NSP10, NSP12 to NSP16, ORF3, ORF6, ORF7a, ORF7b, ORF8, and ORFs for M, E, and N. The SARS-CoV-2 proteins were expressed with the expected molecular sizes, as confirmed by probing HEK293T cells with anti-HA antibody (see Fig. S1A in the supplemental material). To screen SARS-CoV-2 proteins that induce host signaling pathways, HEK293T cells were transfected with a plasmid expressing an individual SARS-CoV-2 protein, along with a plasmid containing a luciferase gene driven by NF-κB (pGL4-NF-κB-LucP2), nuclear factor of activated T-cells (pGL4-NFAT-LucP2), CRE (pGL4-CRE-LucP2), activator protein 1 (AP1) (pGL4-AP1-LucP2), activating transcription factor 6 (ATF6) (pGL4-ATF6-LucP2), SMAD (pGL4-SBE-LucP2), or STAT3 (pGL4-STAT3-LucP2), along with a control plasmid, pNull-RLuc, to normalize transfection efficiency. An empty vector was used as the control. At 24 h after transfection, the level of luciferase activity was measured to determine the ability of viral genes to modify cell signals. We found that the exogenous expression of SARS-CoV-2 NSP6 and ORF7a activated NF-κB signaling almost as efficiently as the positive control, TNF-α (Fig. 1A). Other cellular signals (NFAT, CRE, AP1, SMAD, and STAT3) were not induced by any of the SARS-CoV-2 genes, with the exception that the ORF8 gene significantly activated ATF6 signaling similarly to tunicamycin (Fig. S1B). The ORF8 gene has been implicated in the unfolded protein response, which is regulated by endoplasmic reticulum (ER) stress sensors, including ATF6, inositol-requiring transmembrane kinase endoribonuclease-1α (IRE1α), and protein kinase RNA-like endoplasmic reticulum kinase (PERK) (41). Here, we focused on NSP6 and ORF7a to clarify the molecular mechanisms of NF-κB activation, which induces certain types of cytokines. To examine the effects of NSP6 and ORF7a on inflammatory responses, we measured the levels of NF-κB-dependent cytokines such as IL-8 and IP-10. As expected, the expression of NSP6 and ORF7a enhanced IL-8 and IP-10 expression (Fig. 1B), which was likely a consequence of the NF-κB activation induced by those CoV-2 proteins. *In vitro* kinase assay showed that immunoprecipitated HA-NSP6 or HA-ORF7a induced phosphorylation of glutathione *S*-transferase (GST)-IκBα (Fig. 1C).

10.1128/mbio.00971-22.1 FIG S1 Screening of SARS-CoV-2 genes for signal activation. (A) HEK293T cells were transfected with the indicated plasmid. At 24 h after transfection, the expression of each SARS-CoV-2 protein was analyzed by Western blotting using anti-HA antibody, as indicated by the asterisks. (B) HEK293T cells were transfected with each SARS-CoV-2 protein expression plasmid, along with the indicated reporter plasmid and pNull-RLuc. The level of luciferase activity was determined at 24 h posttransfection. The firefly luciferase activity was normalized to *Renilla* luciferase activity. An empty plasmid was used as a control and set to 1. Treatment of each ligand for 24 h was performed as the positive control. Results are shown as the mean ± SD of three independent experiments. Download FIG S1, TIF file, 2.9 MB. Copyright © 2022 Nishitsuji et al. 2022 Nishitsuji et al. https://creativecommons.org/licenses/by/4.0/ This content is distributed under the terms of the Creative Commons Attribution 4.0 International license.

### NEMO and TAK1 are essential for NF-κB activation by NSP6 and ORF7a.

**FIG 2:**
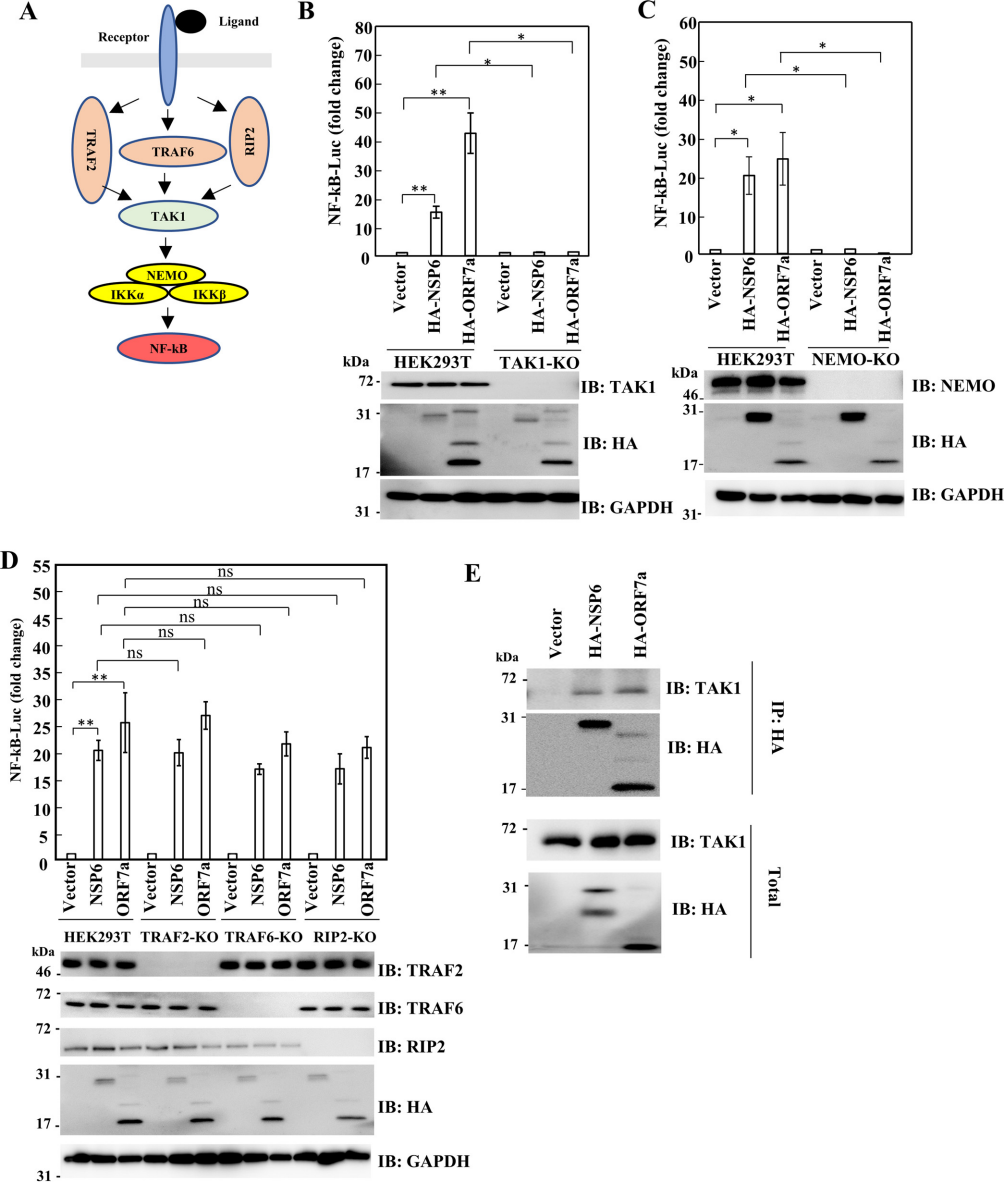
NEMO and TAK1 were necessary for NSP6- and ORF7a-mediated NF-κB activation. (A) Scheme of canonical NF-κB pathway. (B to D) HEK293T-TAK1-KO (B), HEK293T-NEMO-KO (C), and HEK293T-TRAF2-KO, HEK293T-TRAF6-KO, and HEK293T-RIP2-KO (D) cells were transfected with pcDNA3.1-HA, pcDNA3.1-HA-NSP6, or pcDNA3.1-HA-ORF7a, along with pGL4-NF-κB-LucP2 and pNull-RLuc. The level of luciferase activity was determined at 24 h posttransfection. The firefly luciferase activity was normalized to *Renilla* luciferase activity. An empty plasmid was used as a control and set to 1. Results are shown as the mean ± SD of three independent experiments. *, *P* < 0.05; **, *P* < 0.01 (Student’s *t* test). (E) HEK293T cells were transfected with pcDNA3.1-HA, pcDNA3.1-HA-NSP6, or pcDNA3.1-HA-ORF7a. At 48 h after transfection, IP was performed by anti-HA antibody. The immunocomplex was analyzed by Western blotting with the indicated antibodies.

Cytokine receptors, TLRs, and NOD-like receptors (NLRs) are stimulated by specific ligands (42), which is followed by downstream molecule-mediated activation of the canonical NF-κB pathway (Fig. 2A). TAK1 and NEMO are essential factors in the canonical NF-κB pathway (43). To elucidate the mechanism by which NSP6 and ORF7a induce NF-κB activation, we knocked out TAK1 and NEMO in HEK293T cells using the CRISPR-Cas9 system. NSP6- and ORF7a-induced NF-κB activation was blocked by knockout (KO) of TAK1 or NEMO (Fig. 2B and C), suggesting that NSP6 and ORF7a induce canonical NF-κB activation by targeting TAK1 or upstream factors thereof. To further clarify how NSP6 and ORF7a promote NF-κB activation, we knocked out components upstream of TAK1, including TRAF2, TRAF6, and RIP2 (29, 44, 45) (Fig. 2A). Knockout of TRAF2, TRAF6, or RIP2 had no effect on NSP6- or ORF7a-induced NF-κB activation (Fig. 2D). Moreover, NF-κB activation by NSP6 or ORF7a was not decreased in TRAF2-, TRAF5-, or RIP1-deficient cells (Fig. S2). Because these results indicated that NSP6 and ORF7a targeted TAK1, we next examined whether NSP6 or ORF7a could bind to TAK1. Immunoprecipitation (IP) analysis revealed that both NSP6 and ORF7a interacted with TAK1 (Fig. 2E).

10.1128/mbio.00971-22.2 FIG S2 TRAF2, TRAF5, and RIP1 were not required for NSP6- or ORF7a-mediated NF-κB activation. HEK293T, HEK293T-RIP1-KO, HEK293T-TRAF2-double knockout (DKO), and HEK293T-TRAF5-DKO cells were transfected with pcDNA3.1-HA, pcDNA3.1-HA-NSP6, or pcDNA3.1-HA-ORF7a, along with pGL4-NF-κB-LucP2 and pNull-RLuc. The level of luciferase activity was determined at 24 h posttransfection. The firefly luciferase activity was normalized to *Renilla* luciferase activity. An empty plasmid was used as a control and set to 1. Results are shown as the mean ± SD of three independent experiments. **, *P* < 0.01 (Student’s *t*-test). Download FIG S2, TIF file, 2.9 MB. Copyright © 2022 Nishitsuji et al. 2022 Nishitsuji et al. https://creativecommons.org/licenses/by/4.0/ This content is distributed under the terms of the Creative Commons Attribution 4.0 International license.

### Conjugation of K63-linked polyubiquitin chains to NSP6 by TRIM13.

**FIG 3:**
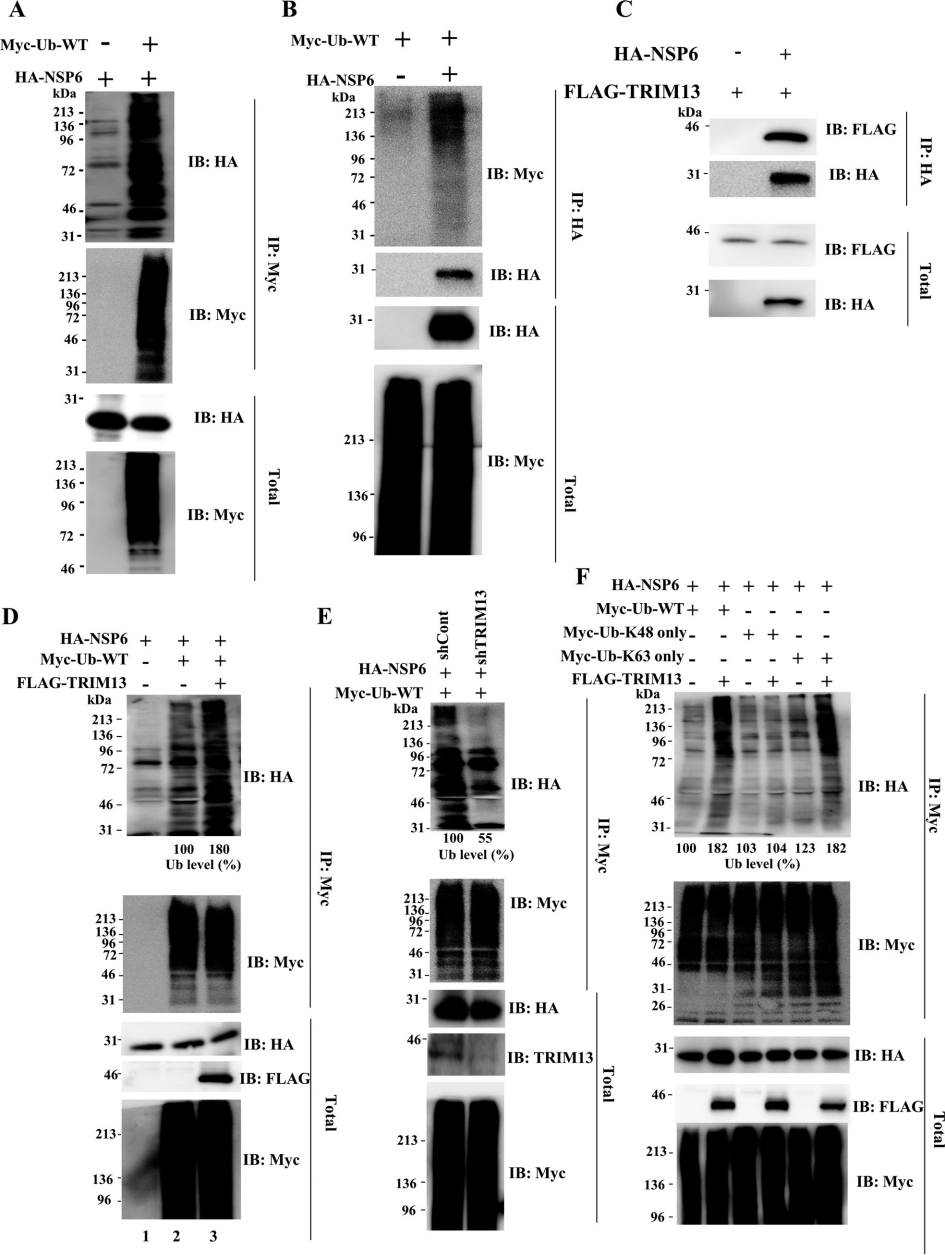
K63-linked NSP6 polyubiquitination by TRIM13. (A and B) HEK293T cells were transfected with the indicated plasmids. At 48 h after transfection, cells were lysed with 1× IP lysis buffer, and the lysates were subjected to ubiquitination assay. (C) HEK293T cells were transfected with the indicated plasmids. At 48 h after transfection, cells were lysed with IP lysis buffer, followed by IP with the anti-HA antibody. The immunocomplex was subjected to Western blotting using the indicated antibodies. (D) HEK293T cells were transfected with the indicated plasmids. At 48 h after transfection, cells were lysed with 1× IP lysis buffer, followed by the ubiquitination assay. The relative level of ubiquitinated NSP6 in the FLAG-TRIM13-transfected cells (lanes 3) was expressed as the percentage of the control cells (lane 2). (E) HEK293T or TRIM13 knockdown HEK293T cells were transfected with the indicated plasmids. At 48 h after transfection, cells were lysed with 1× IP lysis buffer, followed by ubiquitination assay. The relative level of ubiquitinated NSP6 in the TRIM13 knockdown cells was expressed as the percentage of the control cells. (F) HEK293T cells were transfected with the indicated plasmids. At 48 h after transfection, cells were lysed with 1× IP lysis buffer, followed by ubiquitination assay. The numerical values below the blots indicate the level of ubiquitinated NSP6.

The host ubiquitination system tightly controls the NF-κB pathway. Interestingly, recent host proteome analyses have revealed that several SARS-CoV-2 proteins are modified by ubiquitin (Ub) (46, 47). To evaluate whether NSP6 could be ubiquitinated, HEK293T cells were transfected with a plasmid expressing HA-NSP6 along with Myc-Ub. At 48 h after transfection, a ubiquitination assay was performed, using the indicated antibodies. We observed that NSP6 was modified by ubiquitin (Fig. 3A and B). To identify the ubiquitin E3 ligase responsible for the ubiquitination of NSP6, we referred to the COVID-19 interactome and Biological General Repository for Interaction Data sets (BioGRID), which are databases of host protein-SARS-CoV-2 protein interactions, and found that TRIM13 may interact with NSP6. Moreover, previous reports indicated that TRIM13 and NSP6 are localized to the endoplasmic reticulum (48, 49). Indeed, IP analysis revealed that NSP6 interacted with TRIM13 (Fig. 3C). To demonstrate whether TRIM13 conjugated polyubiquitin chains, HEK293T cells were transfected with a control plasmid or plasmid expressing FLAG-TRIM13 and then subjected to the ubiquitination assay. The polyubiquitination level of NSP6 was higher in TRIM13-overexpressed HEK293T cells than in control cells (Fig. 3D). In contrast, knockdown of TRIM13 reduced the polyubiquitination level of NSP6, suggesting that TRIM13 can ubiquitinate NSP6 (Fig. 3E).

To further determine which lysine residues of ubiquitin are required for TRIM13-mediated polyubiquitination of NSP6, we used Myc-Ub-K48 only or Myc-Ub-K63 only (all lysines were mutated to arginine except K48 and K63) as a substrate of ubiquitination. Polyubiquitination of NSP6 was not increased by TRIM13 when Myc-Ub-K48 only was transfected (Fig. 3F) but was increased by TRIM13 in the presence of Myc-Ub-K63 only (Fig. 3F). These results suggest that K63-linked ubiquitination was the primary mode of TRIM13-mediated NSP6 polyubiquitination, although K48-linked polyubiquitination may still occur (Fig. 3F).

### Importance of NSP6 polyubiquitination by TRIM13 for NF-κB activation.

**FIG 4:**
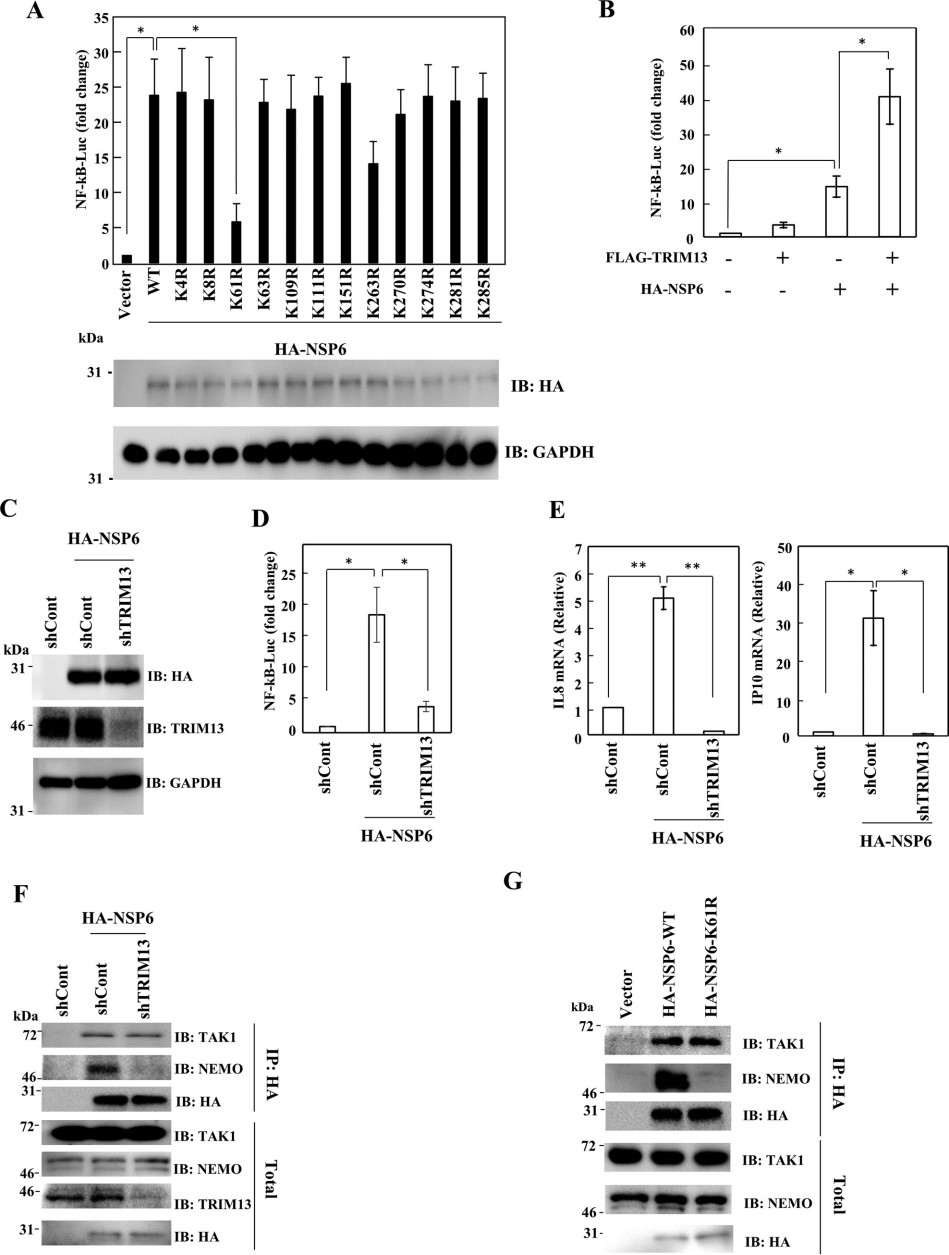
TRIM13 was required for NSP6-mediated NF-κB activation. (A) HEK293T cells were transfected with pcDNA3.1-HA, pcDNA3.1-HA-NSP6, or indicated NSP6 variants, along with pGL4-NF-κB-LucP2 and pNull-RLuc. The level of luciferase activity was determined at 24 h posttransfection. The firefly luciferase activity was normalized to *Renilla* luciferase activity. An empty plasmid was used as a control and set to 1. Results are shown as the mean ± SD of three independent experiments. *, *P* < 0.05 (Student's *t* test). (B) HEK293T cells were transfected with pcDNA-HA, pcDNA3.1-HA-NSP6, or pcDNA3-FLAG-TRIM13, along with pGL4-NF-κB-LucP2 and pNull-RLuc. The level of luciferase activity was determined at 24 h posttransfection. The firefly luciferase activity was normalized to *Renilla* luciferase activity. An empty plasmid was used as a control and set to 1. Results are shown as the mean ± SD of three independent experiments. *, *P* < 0.05 (Student’s *t* test). (C to E) HEK293T or TRIM13 knockdown HEK293T cells were transfected with the indicated plasmid. (C) At 48 h after transfection, cells were subjected to Western blotting. NF-κB reporter assay (D) and RT-qPCR (E). Results are shown as the mean ± SD of three independent experiments. *, *P* < 0.05; **, *P* < 0.01 (Student’s *t* test). (F) HEK293T cells or TRIM13-knockdown HEK293T cells were transfected with the indicated plasmids. At 48 h after transfection, cells were lysed with IP lysis buffer, followed by IP using anti-HA antibody. The immunocomplex was subjected to Western blotting using the indicated antibodies. (G) HEK293T cells were transfected with the indicated plasmids. At 48 h after transfection, cells were lysed with IP lysis buffer, followed by IP using anti-HA antibody. The immunocomplex was subjected to Western blotting using the indicated antibodies.

The SARS-CoV-2 NSP6 protein has 12 lysine residues that may be conjugated with ubiquitin. To map the polyubiquitinated lysine residues of NSP6, we constructed mutants harboring arginine in individual lysine residues in NSP6 (designated NSP6 K#R, where # indicates the amino acid number of the mutated lysine residue) and then performed the ubiquitination assay in the absence or presence of TRIM13. Polyubiquitination of NSP6 K61R was not increased by TRIM13, while the wild-type (WT) and other KR mutants all exhibited increased ubiquitination (Fig. S3) as shown previously (Fig. 3D). Therefore, the lysine 61 residue of NSP6 is essential for TRIM13-mediated polyubiquitination. To determine whether the lysine 61 residue of NSP6 was necessary for NF-κB activation, reporter assays were conducted using WT or KR mutants of NSP6. NSP6-K61R, but not other mutants, showed significantly reduced NF-κB activation compared with NSP6-WT (Fig. 4A), suggesting that TRIM13-mediated polyubiquitination of NSP6 at lysine 61 is important for NF-κB activation.

10.1128/mbio.00971-22.3 FIG S3 K61 of NSP6 was a critical residue for TRIM13-mediated NSP6 polyubiquitination. HEK293T cells were transfected with the indicated plasmids. At 48 h posttransfection, cells were lysed with 1× IP lysis buffer, followed by the ubiquitination assay. Download FIG S3, TIF file, 2.9 MB. Copyright © 2022 Nishitsuji et al. 2022 Nishitsuji et al. https://creativecommons.org/licenses/by/4.0/ This content is distributed under the terms of the Creative Commons Attribution 4.0 International license.

TRIM13 functions as an E3 ligase for TRAF6 ubiquitin conjugation to promote NF-κB activity and thus induces the activation of TLR2-mediated immune responses (50). On the other hand, TRIM13 suppresses proinflammatory responses to viral DNAs (51). To evaluate the physiological roles of TRIM13-mediated NSP6 ubiquitination in NF-κB activation, we examined NF-κB activity in the context of simultaneous expression of TRIM13 and NSP6. Coexpressing TRIM13 with NSP6 enhanced NF-κB activation compared with NSP6 alone (Fig. 4B). Furthermore, we knocked down TRIM13 by short hairpin RNA (shRNA) to clarify the function of endogenous TRIM13. An shRNA targeting TRIM13 suppressed the expression of TRIM13 (Fig. 4C). As expected, NSP6-mediated NF-κB activation was significantly reduced by knockdown of TRIM13 (Fig. 4D). The effect of TRIM13 was further confirmed by the IL-8 and IP-10 mRNA levels (Fig. 4E), suggesting that polyubiquitination of NSP6 by TRIM13 is critical for NF-κB activation.

Next, we investigated the role of TRIM13 in the binding of NSP6 to TAK1 and found no effect thereon of TRIM13 knockdown (Fig. 4F). Interestingly, however, NSP6 failed to recruit NEMO when TRIM13 was knocked down, while NEMO was coprecipitated in the control shRNA (shControl) (Fig. 4F). We also found that when the lysine 61 of NSP6 was swapped with arginine, it could no longer associate with NEMO (Fig. 4G). These results suggest that polyubiquitination of NSP6 at K61 is essential for the recruitment of NEMO.

We also evaluated the role of ubiquitin conjugation to NEMO. To this end, NEMO-KK285,309RR, a mutant that fails to conjugate linear polyubiquitination (52), was prepared, followed by reporter assays. NEMO knockout cells were transfected with NEMO-WT or NEMO-KK285,309RR, along with the indicated plasmids. Consistent with Fig. 2C, NSP6 did not induce NF-κB activation in NEMO-deficient cells (Fig. S4A). Ectopic expression of NEMO-WT rescued the NF-κB activation mediated by NSP6 in NEMO-deficient cells. However, NSP6 failed to induce NF-κB activation in NEMO-deficient cells expressing NEMO-KK285,309RR. In addition, NEMO-F312A, which lacks the ability to bind to polyubiquitin chains (53), also could not rescue NF-κB activation. Intriguingly, NSP6 expression, but not NSP6-K61R, increased the ubiquitin level of NEMO (Fig. S4B). Taken together, both polyubiquitination and polyubiquitin binding of NEMO are important for NSP6-mediated NF-κB activation.

10.1128/mbio.00971-22.4 FIG S4 Effects of NEMO mutations on NSP6- and ORF7a-dependent NF-κB activation. (A) HEK293T-NEMO-KO cells were transduced with enhanced green fluorescent protein (EGFP), NEMO-WT, NEMO-KK285,309RR, or NEMO-F312A using the lentiviral vector system. These cells were transfected with the indicated plasmid, along with pGL4-NF-κB-LucP2 and pNull-RLuc. The level of luciferase activity was determined at 24 h posttransfection. The firefly luciferase activity was normalized to *Renilla* luciferase activity. An empty plasmid was used as a control and set to 1. Results are shown as the mean ± SD of three independent experiments. *, *P* < 0.05; **, *P* < 0.01 (Student’s *t* test). (B) HEK293T cells were transfected with the indicated plasmids. At 48 h after transfection, cells were lysed with 1× IP lysis buffer, followed by ubiquitination assay. LC and NS indicate light chain and nonspecific, respectively. (C) HEK293T cells were transduced with the indicated lentiviral vector expressing shRNA. At 48 h after transduction, cells were transfected with the indicated plasmid, along with pGL4-NF-κB-LucP2 and pNull-RLuc. The level of luciferase activity was determined at 24 h posttransfection. The firefly luciferase activity was normalized to *Renilla* luciferase activity. An empty plasmid was used as a control and set to 1. Results are shown as the mean ± SD of three independent experiments. **, *P* < 0.01 (Student’s *t* test). Download FIG S4, TIF file, 2.9 MB. Copyright © 2022 Nishitsuji et al. 2022 Nishitsuji et al. https://creativecommons.org/licenses/by/4.0/ This content is distributed under the terms of the Creative Commons Attribution 4.0 International license.

### RNF121 is required for the ubiquitination of ORF7a, which activates NF-κB.

**FIG 5:**
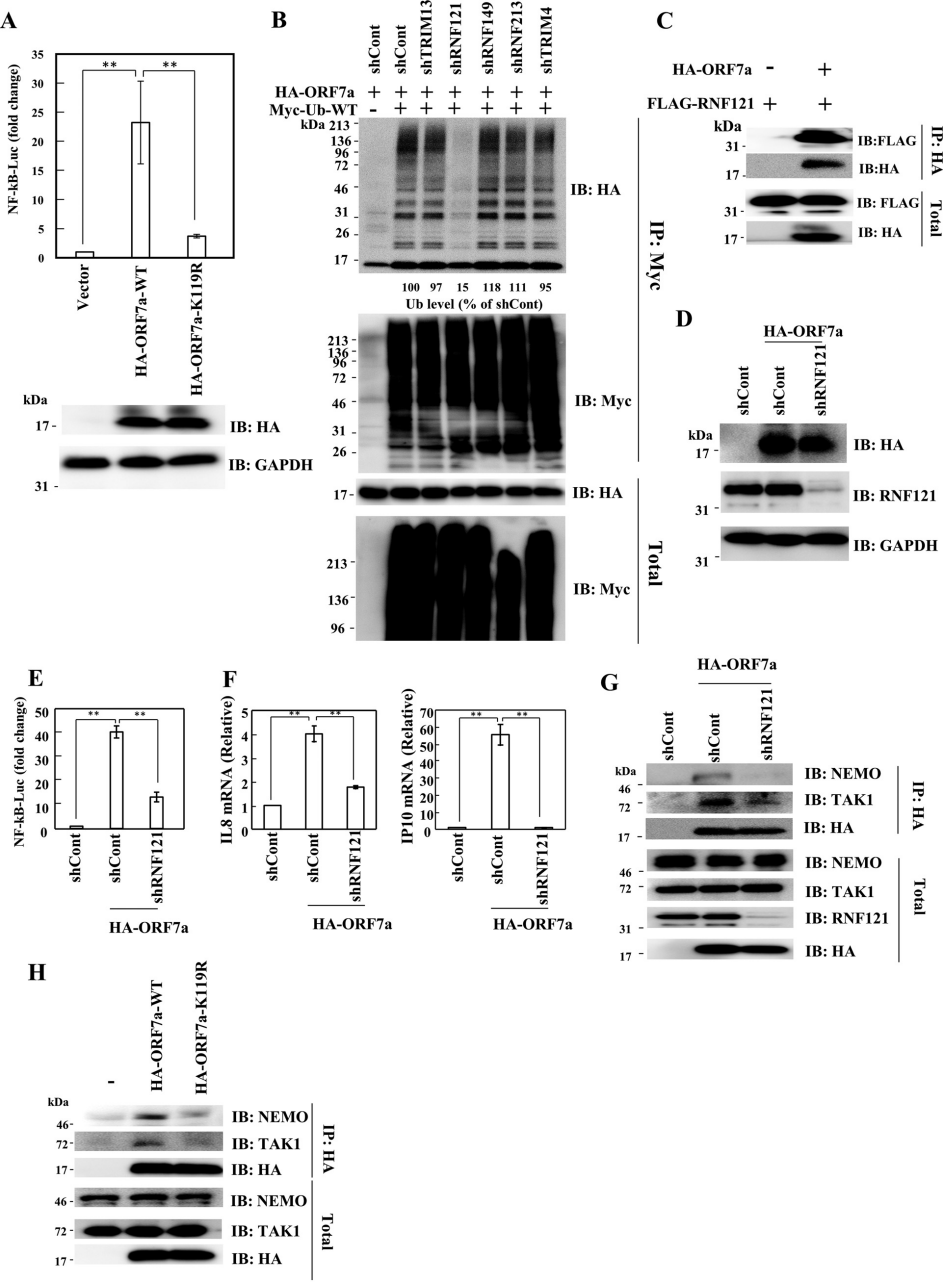
Importance of ORF7a ubiquitination by RNF121 for NF-κB activation. (A) HEK293T cells were transfected with pcDNA3.1-HA, pcDNA3.1-HA-ORF7a, or pcDNA3.1-HA-ORF7a-K119R, along with pGL4-NF-κB-LucP2 and pNull-RLuc. The level of luciferase activity was determined at 24 h posttransfection. The firefly luciferase activity was normalized to *Renilla* luciferase activity. An empty plasmid was used as a control and set to 1. Results are shown as the mean ± SD of three independent experiments. **, *P* < 0.01 (Student’s *t* test). (B) HEK293T cells were transduced with the indicated lentiviral vector expressing shRNA. At 48 h postransduction, cells were transfected with the indicated plasmids. Cells were lysed with 1× IP lysis buffer at 24 h posttransfection, followed by ubiquitination assay. The numerical values below the blots indicate the levels of ubiquitinated ORF7a. (C) HEK293T cells were transfected with the indicated plasmids. At 48 h posttransfection, cells were lysed with IP lysis buffer, followed by IP using anti-HA antibody. The immunocomplex was subjected to Western blotting using the indicated antibodies. (D to F) HEK293T or RNF121-knockdown 293T cells were transfected with the indicated plasmid. At 48 h posttransfection, cells were subjected to Western blotting (D), NF-κB reporter assay (E), or RT-qPCR (F). Results are shown as the mean ± SD of three independent experiments. **, *P* < 0.01 (Student’s *t* test). (G) HEK293T cells or RNF121-knockdown HEK293T cells were transfected with the indicated plasmids. At 48 h posttransfection, cells were lysed with IP lysis buffer, followed by IP using anti-HA antibody. The immunocomplex was subjected to Western blotting using the indicated antibodies. (H) HEK293T cells were transfected with the indicated plasmids. At 48 h posttransfection, cells were lysed with IP lysis buffer, followed by IP using anti-HA antibody. The immunocomplex was subjected to Western blotting using the indicated antibodies.

SARS-CoV-2 ORF7a at lysine 119 is conjugated to K63-linked ubiquitin chains and inhibits type I IFN signaling (54). Therefore, we determined whether K119 of ORF7a affects NF-κB activation. When 293T cells were transfected with HA-ORF7a-WT, NF-κB activation was significantly enhanced compared with empty plasmid (Fig. 5A). This effect was severely impaired by a single point mutation of K119 to R (K119R). Because the E3 ubiquitin ligases that regulate the polyubiquitination of ORF7a are unknown, we searched for an E3 ubiquitin ligase that modifies ORF7a. Databases of host protein-SARS-CoV-2 protein interactions and BioGRID suggested that ORF7a likely binds to TRIM13, RNF149, RNF213, and TRIM4. Knockdown studies showed that these genes had no effect on the polyubiquitination of ORF7a (Fig. S5 and Fig. 5B). Because ORF7a is localized in the ER-Golgi (55), we further searched for an E3 ubiquitin ligase localized to that compartment, and we identified RNF121 (56). A previous report has indicated that RNF121 regulates NF-κB activation through an unknown mechanism (57). Knockdown of RNF121 reduced the polyubiquitination of ORF7a (Fig. S5 and Fig. 5B). Moreover, the IP assay revealed that ORF7a interacted with RNF121 (Fig. 5C), suggesting that RNF121 plays a critical role in the polyubiquitination of ORF7a. Next, we examined whether RNF121 regulates ORF7a-dependent NF-κB activation. As expected, ORF7a-mediated NF-κB reporter activation was significantly blocked by shRNF121 compared with shControl (Fig. 5D and E). Accordingly, the mRNA levels of IL-8 and IP-10 were decreased by knockdown of RNF121 in the presence of ORF7a, indicating that RNF121-mediated ubiquitination of ORF7a is required for NF-κB activation (Fig. 5F).

10.1128/mbio.00971-22.5 FIG S5 Confirmation of knockdown of possible E3 ligases. HEK293T cells were transduced with the indicated lentiviral vector expressing shRNA. At 48 h after transduction, the level of indicated genes was determined by RT-qPCR. The indicated mRNA levels were normalized to GAPDH expression. Control shRNA was used as a control and set to 1. Results are shown as the mean ± SD of three independent experiments. **, *P* < 0.01 (Student’s *t*-test). Download FIG S5, TIF file, 1.9 MB. Copyright © 2022 Nishitsuji et al. 2022 Nishitsuji et al. https://creativecommons.org/licenses/by/4.0/ This content is distributed under the terms of the Creative Commons Attribution 4.0 International license.

To elucidate the role of RNF121 in the binding of ORF7a to NEMO and TAK1, we performed an IP assay. ORF7a coimmunoprecipitated NEMO and TAK1 in shControl cells, but knockdown of RNF121 reduced the binding of ORF7a to NEMO and TAK1 (Fig. 5G). Moreover, ORF7a-K119R could not interact with TAK1 and NEMO as efficiently as ORF7a-WT (Fig. 5H). To further determine how ORF7a activates the NF-κB pathway through NEMO, NEMO-WT, NEMO-KK285,309RR, or NEMO-F312A was introduced into NEMO-deficient cells (Fig. S4A). Ectopic expression of NEMO-WT and NEMO-KK285,309RR restored NF-κB activation by ORF7a. However, ORF7a-induced NF-κB activation was still low in NEMO-deficient cells expressing NEMO-F312A. These results suggest that RNF121-mediated polyubiquitination of ORF7a is required for the recruitment of NEMO and TAK1 to ORF7a.

### TAB2 and TAB3 are required for ORF7a-mediated NF-κB activation.

The TAK1/TAB1/TAB2/TAB3 complex phosphorylates IKKs and then stimulates the NF-κB pathway (58, 59). To determine the role of TAB1, TAB2, and TAB3 in NF-κB activation mediated by NSP6 and ORF7a, TAB1, TAB2, and TAB3 were knocked down in HEK293T cells (Fig. S4C). Silencing of TAB1 had no effect on NSP6- or ORF7a-triggered NF-κB activation. This result is consistent with a previous report that TAB1 is not required for NF-κB activation after treatment with TNF-α or IL-1 (60). Single or synchronous knockdown of TAB2 and/or TAB3 had no effect on the NF-κB activation by NSP6. However, NF-κB activation by ORF7a was marginally decreased by knockdown of TAB2 or TAB3 alone and significantly decreased by simultaneous knockdown of TAB2 and TAB3. These results suggest that ubiquitinated ORF7a may require the K63-linked polyubiquitin-binding activity of TAB2 and TAB3 (59) to bind TAK1 for NF-κB activation. Since ORF7a requires its polyubiquitination to bind to TAK1 (Fig. 5G and H), K63-linked polyubiquitin binding activity of TAB2 and TAB3 in the TAK1-TABs complex (59) plays an important role in the binding of ORF7a to TAK1 and ORF7a-mediated NF-κB activation. In contrast, TAB2 and TAB3 may not be essential for NSP6-mediated NF-κB activation because NSP6 can bind to TAK1 in a ubiquitin-independent manner.

### Induction of IL-8, IP-10, IL-1β, and IL-6 by SARS-CoV-2 infection is mediated by TRIM13 and RNF121.

**FIG 6:**
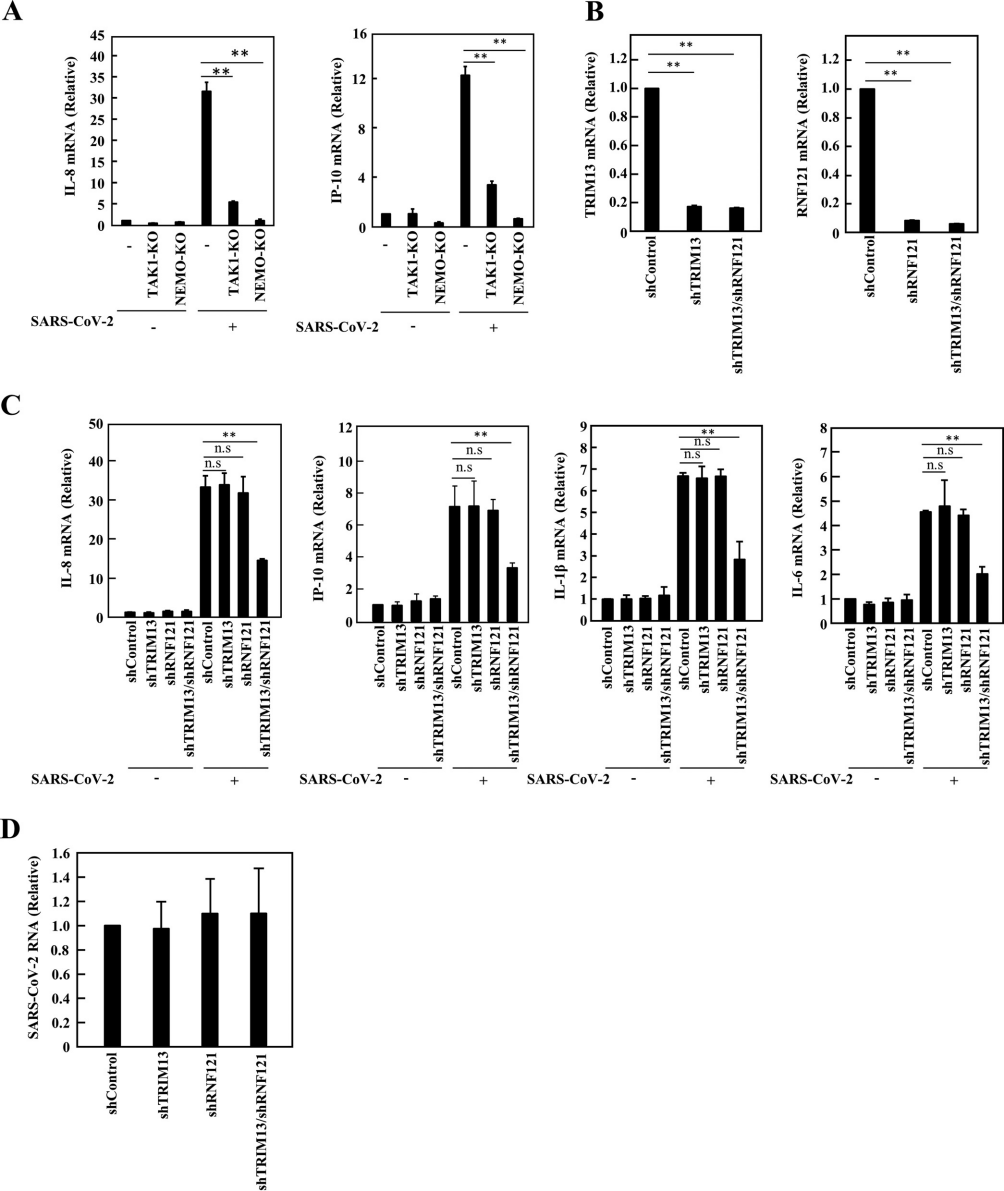
Both TRIM13 and RNF121 are required for IL-8, IP-10, IL-1β, and IL-6 induction in SARS-CoV-2-infected cells. (A) HEK293T-ACE2, HEK293T-TAK1-KO-ACE2, or HEK293T-NEMO-KO-ACE2 cells were infected with SARS-CoV-2. At 24 h after infection, the level of IL-8 and IP-10 was determined by RT-qPCR. The indicated mRNA levels were normalized to GAPDH expression. (B to D) HEK293T-ACE2 cells were transduced with the indicated lentiviral vector expressing shRNA. At 48 h after transduction, cells were infected with SARS-CoV-2. At 24 h after infection, the levels of TRIM13, RNF121 (B), IL-8, IP-10, IL-1β, IL-6 (C), and SARS-CoV-2 RNA (D) were determined by RT-qPCR. The indicated mRNA levels were normalized to GAPDH expression. Results are shown as the mean ± SD of three independent experiments. **, *P* < 0.01 (Student’s *t* test).

To investigate whether TAK1 and NEMO have an important role in NF-κB activation by SARS-CoV-2 infection, TAK1 or NEMO knockout HEK293T cells expressing ACE2 were infected with SARS-CoV-2 for 24 h, and the levels of IL-8 and IP-10 mRNAs were measured. Consistent with a previous report (21), SARS-CoV-2 infection could lead to the expression of IL-8 and IP-10 compared with noninfection control (Fig. 6A). Importantly, induction of IL-8 and IP-10 was severely reduced by knockout of TAK1 or NEMO. To next determine the physiological relevance of TRIM13 and RNF121 in SARS-CoV-2-induced inflammatory responses, we silenced TRIM13 and RNF121 in HEK293T-ACE2 cells (Fig. 6B) and determined the levels of IL-8, IP-10, IL-1β and IL-6 mRNAs in SARS-CoV-2-infected cells (Fig. 6C). Depletion of either TRIM13 or RNAF121 had no effect on the expression of IL-8, IP-10, IL-1β, and IL-6 in SARS-CoV-2-infected cells. However, the levels of IL-8, IP-10, IL-1β, and IL-6 were significantly reduced when both the TRIM13 and RNF121 genes were silenced together compared with control cells after SARS-CoV-2 infection, suggesting that both TRIM13 and RNF121 are required for the inflammatory response to SARS-CoV-2 infection. In addition, depletion of TRIM13 and/or RNAF121 had no effect on the level of SARS-CoV-2 RNA (Fig. 6D). Collectively, these results show that the ubiquitination of NSP6 and ORF7a is critical for the potent induction of inflammatory cytokines mediated by SARS-CoV-2 infection.

## DISCUSSION

SARS-CoV-2 infection exhibits a weaker type I IFN response than other common respiratory viruses, including SARS-CoV-1, Middle East respiratory syndrome-CoV, respiratory syncytial virus (RSV), and influenza A virus (9). Several SARS-CoV-2 proteins can suppress the type I IFN signaling pathway at multiple steps, including the recognition of viral RNA (16, 61), ubiquitination of NEMO (62), and phosphorylation of TBK1 (63), IRF3 (64), and STAT1/2 (65, 66). The reduced antiviral type I IFN response must be beneficial for efficient replication of SARS-CoV-2. In contrast, the robust proinflammatory response induced by SARS-CoV-2 infection is a cause of cytokine storm, resulting in severe COVID-19 symptoms, including organ damage and acute respiratory distress syndrome (7, 67–69). The serum levels of IL-2, IL-6, TNF-α, IL-1β, IL-10, IFN-γ, IL-8, and IP-10 are significantly increased in patients with severe COVID-19 (70), possibly via selective activation of NF-κB. These studies led us to propose the following model. Multiple SARS-CoV-2 proteins restrict the type I IFN response to evade host immunity, and they extend its propagation in the early stage. In the later stage, when infected individuals suffer from severe symptoms, activation of NF-κB by viral components, including viral RNA and proteins, becomes prominent, leading to cytokine storm in severe COVID-19 cases (71). Therefore, understanding the molecular mechanism by which SARS-CoV-2 infection induces proinflammatory cytokines is important for protection from, and treatment of, severe COVID-19. In this study, SARS-CoV-2 NSP6 and ORF7a induced proinflammatory cytokines, such as IL-8 and IP-10, through the activation of NF-κB.

We propose the following working hypothesis. During NF-κB activation by NSP6 (Fig. S6A), NSP6 interacts with TAK1 in a ubiquitination-independent manner, TRIM13 mediates K63-linked polyubiquitination at NSP6-K61, and the IKK complex is then recruited by K63-linked polyubiquitination of NSP6. TAK1 stimulates the IKK complex, leading to the ubiquitination of NEMO, and ubiquitinated NEMO further recruits the IKK complex, resulting in NF-κB activation. In this model, ubiquitinated NSP6 plays an important role as a “bridge” to recruit the IKK complex and conjugate ubiquitin chains to NEMO. Indeed, our result revealed that wild-type NSP6, but not the polyubiquitination-defective mutant of NSP6 (NSP6-K61R), facilitated polyubiquitination of NEMO (Fig. S4B), resulting in transactivation between IKK complexes that led to NF-κB activation.

10.1128/mbio.00971-22.6 FIG S6 Schematic model of NSP6- and ORF7a-mediated NF-κB activation. (A) Working hypothesis of NF-κB activation by NSP6. (A, panel 1) NSP6 associates with TAK1 and TRIM13, which induces the K63-linked polyubiquitination of NSP6. In turn, this induces recruitment and transphosphorylation of the IKK complex. NEMO is further ubiquitinated for more efficient recruitment and activation of the IKK complex. Activation of IKK results in the phosphorylation and ubiquitin-dependent degradation of IκB, which causes the release and activation of the canonical NF-κB transcription factors, p50 and p65. (A, panel 2) NSP6-K61R, a ubiquitination-defective mutant of NSP6, cannot recruit the IKK complex because NEMO cannot bind to NSP6-K61R (Fig. 4G). (A, panel 3) NEMO-KK285,309RR, a ubiquitination-defective mutant of NEMO, cannot induce transactivation of the IKK complex. (A, panel 4) NEMO-F312A, a defective mutant associated with the binding activity of NEMO to ubiquitin, cannot be activated by TAK1. (B, panel 1) ORF7a interacts with RNF121, and RNF121 mediates the polyubiquitination of ORF7a. TAK1 and the IKK complex are recruited to the scaffold of the polyubiquitin, and the IKK complex is activated by TAK1, resulting in transactivation between IKK complexes. (B, panel 2) ORF7a-K119R, a ubiquitination-defective mutant of ORF7a, cannot recruit IKK complexes. (B, panel 3) NEMO-KK285,309RR can induce NF-κB activation, as mediated by ORF7a. Unlike the activation by NSP6, further ubiquitination of NEMO is not needed for efficient activation of NF-κB signaling by ORF7a. (B, panel 4) Ubiquitinated ORF7a recruits TAK1, while TAK1 cannot induce the activation of NEMO-F312A. Download FIG S6, TIF file, 2.4 MB. Copyright © 2022 Nishitsuji et al. 2022 Nishitsuji et al. https://creativecommons.org/licenses/by/4.0/ This content is distributed under the terms of the Creative Commons Attribution 4.0 International license.

Regarding NF-κB activation by ORF7a (Fig. S6B), RNF121 induces the polyubiquitination of ORF7a; polyubiquitinated ORF7a also appears to function as a scaffold for recruiting TAK1 and IKK complexes because inhibition of ORF7a ubiquitination by RNF121 knockdown or K119R mutation of ORF7a disrupted ORF7a binding to not only NEMO, but also TAK1 (Fig. 5G and H), and repressed NF-κB activation as well (Fig. 5A). Polyubiquitination of NEMO itself is not required for NF-κB activation by ORF7a, unlike that induced by NSP6 (Fig. S4A); however, the binding of NEMO to polyubiquitin chains is required for ORF7a-induced NF-κB activation (Fig. S4A).

Although NF-κB activation by solitary expression of NSP6 or ORF7a was severely reduced by silencing of TRIM13 or RNF121, respectively (Fig. 4E and Fig. 5F), NF-κB activation by SARS-CoV-2 infection remained by TRIM13 and RNF121 double knockdown (Fig. 6C), though more than 50% reduction of NF-κB activation was observed. This may be explained by the alteration of the biological function of those proteins under coexisting conditions with other virus proteins that may reflect more physiological features of those proteins in infected cells. Another possibility is that some molecule other than NSP6 and ORF7a may induce NF-κB activation. In fact, the liquid-liquid phase separation (LLPS) of SARS-CoV-2 N protein promotes the expression of proinflammatory cytokines through NF-κB hyperactivation (36). This LLPS is observed in the association of N protein with specific virus RNA, but not any of host RNAs (72). The fact that we did not observe N protein-mediated NF-κB activation might be due to the absence of the virus RNA in our experimental condition (Fig. 1A). SARS-CoV-2 N protein is an RNA-binding protein that plays a role in the selective packaging of viral RNA. The LLPS of N protein is triggered by specific viral RNA sequences. TAK1 and IKKβ are recruited by the LLPS of N protein, resulting in NF-κB activation (36). It seems possible that the SARS-CoV-2 N protein without association with viral RNAs does not lead to induce NF-κB activation. Moreover, SARS-CoV-2 RNA is sensed by pattern recognition receptors such as RIG-I or MDA5, which induce proinflammatory cytokines (10).

Both TRIM13 and RNF121 are E3 ligases that are localized at the ER. Since SARS-CoV-2 infection induces ER stress (41), SARS-CoV-2 infection may regulate the function of these proteins by unknown mechanisms. Indeed, the level of RNF121 increases after ER stress (73), which may result in induced polyubiquitination of ER-localized ORF7a. Further studies are needed to clarify how these proteins are regulated under the inflammation conditions upon SARS-CoV-2 infection.

Importantly, NF-κB activation by SARS-CoV-2 infection is dramatically reduced in TAK1 or NEMO knockout cells (Fig. 6A). It is still noteworthy that other virus proteins than those described here and/or virus RNA may have an additional role for the upregulation of cytokines, such as IL-8 and IP-10, through activation of NF-κB. It remains to be further analyzed. However, whatever the unknown virus proteins that contribute to NF-κB activation, TAK1 and NEMO may be potential novel therapeutic targets for COVID-19.

Inhibition of NF-κB signaling, using various inhibitors or siRNA (targeting p65), suppressed not only the production of proinflammatory cytokines but also SARS-CoV-2 replication (21); this suggests that SARS-CoV-2 hijacks the NF-κB signaling pathway for efficient multiplication. Therefore, suppressing proinflammatory cytokine responses by NF-κB, while providing sufficient immunity for viral clearance, could be a useful treatment approach for COVID-19 (74).

## MATERIALS AND METHODS

### Cells.

HEK293T and Vero cells were maintained in Dulbecco’s modified Eagle’s medium (catalog no. 08458-16; Nacalai Tesque, Inc., Kyoto, Japan) supplemented with 10% heat-inactivated fetal bovine serum (catalog no. SH30088.03; Cytiva, Marlborough, MA, USA), 100 U/mL penicillin-100 μg/mL streptomycin (catalog no. 26253-84; Nacalai Tesque), and 1% nonessential amino acids solution (catalog no. 11140050; Thermo Fisher Scientific, Waltham, MA, USA) at 37°C and 5% CO_2_.

### Antibodies.

The antibodies used in this study were as follows: anti-TRAF2 (1:1,000; catalog no. 4724; Cell Signaling Technology [CST], Danvers, MA, USA), anti-TRAF6 (1:1,000; catalog no. 8028; CST), anti-RIP2 (1:1,000; catalog no. 4142; CST), anti-TAK1 (1:1,000; catalog no. 5206; CST), anti-IKKγ (1:1,000; catalog no. 2685; CST), anti-HA (1:2,000; catalog no. 3724; CST), anti-TAB1 (1:2,000; catalog no. 3226; CST), anti-TAB2 (1:2,000; catalog no. 3745; CST), anti-TAB3 (1:2,000; catalog no. 14241; CST), anti-IKKβ (1:1,000; catalog no. 8943; CST), anti-Myc tag (1:2,000; catalog no. 2278; CST), anti-GAPDH (glyceraldehyde-3-phosphate dehydrogenase; 1:5,000; catalog no. 39-8600; Thermo Fisher Scientific), anti-RNF121 (1:1,000; catalog no. HPA046041; Sigma-Aldrich, St. Louis, MO, USA), anti-TRIM13 (1:1,000; catalog no. HPA000367; Sigma-Aldrich), horseradish peroxidase (HRP)-conjugated anti-mouse IgG (1:3,000; catalog no. AP308P; Sigma-Aldrich), anti-FLAG M2 antibody produced in mouse (1:1,000; catalog no. F3165; Sigma-Aldrich), anti-FLAG antibody produced in rabbit (1:1,000; catalog no. SAB4301135; Sigma-Aldrich), anti-GST-tag antibody (1:2,000; catalog no. 2624; CST), anti-thiophosphate ester antibody (1:2,000; catalog no. ab92570; Abcam), and HRP-conjugated anti-rabbit IgG (1:5,000; catalog no. AP187P; Sigma-Aldrich). Anti-HA magnetic beads were purchased from Thermo Fisher Scientific (catalog no. 88837), and anti-Myc tag beads were purchased from Medical & Biological Laboratories (catalog no. 3305; Aichi, Japan).

### Plasmids.

To construct N-terminal HA-tagged SARS-CoV-2 NSP1, NSP2, NSP5, NSP6, NSP8, NSP9, NSP15, ORF3, ORF7b, M, E, and N, total RNA was isolated from SARS-CoV-2-infected Vero cells using the RNeasy Plus minikit (catalog no. 74136; Qiagen, Hilden, Germany). Each gene was amplified by reverse transcription (RT) (catalog no. FSQ-201; Toyobo, Osaka, Japan) and PCR (catalog no. KMM-101; Toyobo) and then inserted into the BamHI and EcoRI sites of pcDNA3.1-HA (75). Nucleotides for other SARS-CoV-2 genes (NSP4, NSP7, NSP10, NSP12, NSP13, NSP14, NSP16, ORF6, ORF7a, and ORF8) were synthesized and cloned into pcDNA-HA after codon optimization (Thermo Fisher Scientific). To generate pcDNA3-FLAG-TRIM13, total RNA was isolated from HEK293T cells. TRIM13 fragment was amplified by RT-PCR and then inserted into the EcoRI and XhoI sites of pcDNA3-FLAG. To construct pcDNA3-FLAG-RNF121, total RNA was isolated from HEK293T cells. The RNF121 fragment was amplified by RT-PCR and then inserted into the EcoRI and XhoI sites of pcDNA3-FLAG. The lentivirus-based shRNA plasmids used in this study were purchased from Sigma and include pLKO.1-puro-shTRIM13 (catalog no. SHCLNG; clone no. TRCN0000271409), pLKO.1-puro-shTRIM4 (catalog no. SHCLNG; clone no. TRCN0000034031), pLKO.1-puro-shRNF121 (catalog no. SHCLNG; clone no. TRCN0000007723), pLKO.1-puro-shRNF149 (catalog no. SHCLNG; clone no. TRCN0000034155), pLKO.1-puro-shRNF213 (catalog no. SHCLNG; clone no. TRCN0000150582), pLKO.1-puro-shTAB1 (catalog no. SHCLNG; clone no. TRCN0000001862), pLKO.1-puro-shTAB2 (catalog no. SHCLNG; clone no. TRCN0000378442), pLKO.1-puro-shTAB3 (catalog no. SHCLNG; clone no. TRCN0000011194), and pLKO.1-puro-shTRIM13 (catalog no. SHCLNG; clone no. TRCN0000271409). The reporter plasmid pGL4-ATF6-LucP2 was purchased from Promega (catalog no. E3661; Madison, WI, USA). To construct the reporter plasmids pGL4-NF-κB-LucP2, pGL4-AP1-LucP2, pGL4-NF-AT-LucP2, pGL4-CRE-LucP2, pGL4-SBE-LucP2, and pGL4-STAT3-LucP2, the ATF6-responsive element of pGL4-ATF6-LucP2 was replaced with each responsive element. pNull-RLuc was previously reported (76). To construct pMyc-Ub, the N-terminal Myc-tagged ubiquitin fragment was inserted into the BamHI-EcoRI sites of pCAG. The ubiquitin mutant plasmids were constructed using the KOD Plus mutagenesis kit (catalog no. SMK-101; Toyobo) according to the manufacturer’s protocol. To generate CRISPR-Cas9-mediated knockout cell lines, MAP3K7 sgRNA CRISPR/Cas9 All-in-One lentivector (catalog no. 280341110595), IKBKG single guide RNA (sgRNA) CRISPR/Cas9 All-in-One lentivector (catalog no. 247991110595), and RIPK2 sgRNA CRISPR/Cas9 All-in-One lentivector (catalog no. 396111110595) were purchased from Applied Biological Materials (Richmond, BC, Canada), and TRAF2 CRISPR/Cas9 KO plasmid (catalog no. sc-400361), TRAF5 CRISPR/Cas9 KO plasmid (catalog no. sc-402073), TRAF6 CRISPR/Cas9 KO plasmid (catalog no. sc-400117), and RIP CRISPR/Cas9 KO plasmid (catalog no. sc-400377) were purchased from Santa Cruz Biotechnology (Dallas, TX, USA). The lentivirus-based ACE2 expression plasmid used in this study was purchased from System Biosciences (catalog no. CVD19-100PA-1; CA, USA).

### Preparation of shRNA-expressing lentivirus vectors.

To generate individual lentivirus vectors, HEK293T cells (5 × 10^5^ cells per well in a 6-well plate) were transfected with individual transfer vector (1 μg), pCAG-HIVgp (0.75 μg), pRSV-Rev (0.25 μg), and pCMV-VSV-G (0.25 μg) using TransIT-293 (catalog no. MIR2700; TaKaRa, Shiga, Japan) according to the manufacturer’s protocol. At 24 h after transfection, culture supernatants were filtered through 0.45-μm-pore-size filters (catalog no. 725-2545; Thermo Fisher Scientific). HEK293T cells were transduced with the individual shRNA lentivirus. At 48 h after transduction, knockdown efficiency was determined by Western blotting or RT-quantitative PCR (RT-qPCR).

### Reporter assays.

HEK293T cells (1 × 10^5^ cells per well in a 24-well plate) were transfected with SARS-CoV-2 protein expression plasmid (400 ng) and pNull-RLuc (50 ng), along with pGL4-ATF6-LucP2 (100 ng), pGL4-NF-κB-LucP2 (5 ng), pGL4-AP1-LucP2 (100 ng), pGL4-NF-AT-LucP2, pGL4-CRE-LucP2 (100 ng), pGL4-SBE-LucP2 (100 ng), or pGL4-STAT3-LucP2 (100 ng) using TransIT-293 according to the manufacturer’s protocol. At 24 h after transfection, cells were harvested and suspended in 0.3 mL passive lysis buffer (catalog no. E153A; Promega). The level of dual-luciferase activity was determined using the dual-luciferase reporter assay system (catalog no. E196; Promega). Luciferase signals were measured by the GloMax Explorer multimode microplate reader (catalog no. GM3600; Promega). Firefly luciferase activities were normalized to *Renilla*, and the fold change was calculated as the ratio of empty plasmid to indicated plasmid.

### IP.

HEK293T cells (5 × 10^5^ cells per well in a 6-well plate) were transfected with the indicated plasmids. At 48 h after transfection, cells were lysed in 0.5 mL IP lysis buffer (10 mM Tris-HCl, pH 7.8, 150 mM NaCl, 1 mM EDTA, and 1% NP-40) supplemented with protease inhibitor cocktail (catalog no. 539134; Merck, Kenilworth, NJ, USA). Cell lysates were centrifuged at 17,700 × *g* for 10 min at 4°C. The supernatant was incubated with 20 μL anti-HA magnetic beads for 2 h at 4°C. The beads were washed five times with IP lysis buffer. The immunocomplex was eluted by boiling with 25 μL of 4× sample buffer (8% sodium dodecyl sulfate [SDS], 20% 2-mercaptoethanol, 40% glycerol, 0.004% bromophenol blue, and 0.25 M Tris-HCl, pH 6.8) and analyzed by Western blotting. To detect the ubiquitination of NSP6 and ORF7a, HEK293T cells (5 × 10^5^ cells per well in a 6-well plate) were transfected with the indicated plasmid. At 48 h after transfection, cells were lysed with 0.5 mL of 1× IP lysis buffer. Cell lysates were denatured at 95°C for 10 min and then sonicated for 10 min. Cell lysates were centrifuged at 17,700 × *g* for 10 min at 4°C. The supernatant was incubated with 20 μL anti-HA magnetic beads or 30 μL anti-Myc-tag beads for 2 h at 4°C. The beads were washed five times with IP lysis buffer. The immunocomplex was eluted by boiling with 25 μL of 4× sample buffer and subjected to Western blotting. Membranes were probed with anti-HA or anti-Myc antibody and horseradish peroxidase-conjugated secondary antibody (catalog no. AP188P; Sigma-Aldrich). Labeled proteins were visualized using a LuminoGraph I (ATTO). Band analysis was performed with ImageSaver6 (ATTO). To detect the ubiquitination of NEMO, HEK293T cells (5 × 10^5^ cells per well in a 6-well plate) were transfected with the indicated plasmid. At 48 h after transfection, cells were lysed with 0.5 mL of 1× IP lysis buffer. Cell lysates were denatured at 95°C for 10 min and then sonicated for 10 min. Cell lysates were centrifuged at 17,700 × *g* for 10 min at 4°C. The supernatant was incubated with 30 μL anti-FLAG M2 antibody mixed with protein G magnetic beads (catalog no. DB10003; Thermo Fisher Scientific) and incubated for 2 h at 4°C. The beads were washed five times with IP lysis buffer. The immunocomplex was eluted by boiling with 25 μL of 4× sample buffer and subjected to Western blotting. Membranes were probed with anti-Myc antibody and then horseradish peroxidase-conjugated secondary antibody. Labeled proteins were visualized using a LuminoGraph I.

### Gene knockout.

HEK293T cells were transfected with the indicated plasmid. At 48 h after transfection, the cells were seeded at a density of 1 cell per well in a 96-well plate. Gene knockout was confirmed by Western blotting.

### RT-qPCR.

HEK293T cells were transfected with the indicated plasmid. At 48 h after transfection, total RNA was isolated using the RNeasy Plus minikit (catalog no. 74136; Qiagen) according to the manufacturer’s protocol. cDNA was prepared using ReverTra Ace (catalog no. FSQ-201; Toyobo) according to the manufacturer’s protocol. qPCR was carried out with PowerUp SYBR Green master mix (catalog no. A25742; Thermo Fisher Scientific), and fluorescence was analyzed with StepOnePlus real-time PCR system (Thermo Fisher Scientific). The data were normalized to GAPDH expression. The primer sequences were as follows: IP10-F, 5′-GTGGCATTCAAGGAGTACCTC-3′; IP10-R, 5′-TGATGGCCTTCGATTCTGGATT-3′; IL-8-F, 5′-CTGTTAAATCTGGCAACCCTAGTCT-3′; IL-8-R, 5′-CAAGGCACAGTGGAACAAGGA-3′; IL-1β-F, 5′-ACAGATGAAGTGCTCCTTCCA-3′; IL-1β-R, 5′-GTCGGAGATTCGTAGCTGGAT-3′; IL-6-F, 5′-AGAGGCACTGGCAGAAAACAAC-3′; IL-6-R, 5′-AGGCAAGTCTCCTCATTGAATCC-3′; SARS-CoV-2-F, 5′-CACATTGGCACCCGCAATC-3′; SARS-CoV-2-R, 5′-GAGGAACGAGAAGAGGCTTG-3′; GAPDH-F, 5′-GCAAATTTCCATGGCACCGT-3′; GAPDH-R, 5′-GCCCCACTTGATTTTGGAGG-3′; TRIM13-F, 5′-TGTGGGATCTGTGCTACTCGTGG-3′; TRIM13-R, 5′-GGAAGAGGGACTCAAAGGCATCC-3′; RNF121-F, 5′-GCACCCACGCTCCTACAATATGG-3′; RNF121-R, 5′-CCAGATCACTAGGAACCTCCACC-3′; RNF149-F, 5′-GGCATGTACAGGAGTTCATCAGC-3′; RNF149-R, 5′-GAGAGCCAGTATATAGGAAACGC-3′; RNF213-F, 5′-CCAAGAAGGAGCTACATCAGAGG-3′; RNF213-R, 5′-GCTTCCTTCTCTTGCTGGGTATG-3′; TRIM4-F, 5′-CACCTCAGGGAAACATTACTGGG-3′; and TRIM4-R, 5′-AATCGCCCAGATGCCCACATCTG-3′.

### SARS-CoV-2 infection.

SARS-CoV-2 (SARS-CoV-2/Hu/DP/Kng/19-020) was obtained from the Kanagawa Prefectural Institute of Public Health, Japan. The virus was propagated in VeroE6/TMPRSS2 cells (JCRB). Two days after infection, the culture medium was centrifuged, and the supernatants were filtrated. This solution was used as the working virus stock.

HEK293T cells were transduced by a lentivirus vector for ACE2 expression. At 48 h after transduction, HEK293T-ACE2 cells were infected with SARS-CoV-2 for 24 h at a multiplicity of infection (MOI) of 2. After infection, the levels of TRIM13, RNF121, IL-8, IP-10, IL-1β, IL-6, and SARS-CoV-2 RNA were determined by RT-qPCR.

### *In vitro* kinase assay.

HEK293T cells were transfected with pcDNA3.1-HA, pcDNA3.1-HA-NSP6, or pcDNA3.1-HA-ORF7a. At 48 h after transfection, cells were lysed in 1.0 mL IP lysis buffer supplemented with protease inhibitor cocktail. Cell lysates were centrifuged at 17,700 × *g* for 10 min at 4°C. The supernatant was incubated with 50 μL anti-HA magnetic beads for 2 h at 4°C. The beads were washed five times with IP lysis buffer and once with kinase buffer (40 mM Tris-HCl, pH 7.5, 20 mM MgCl_2_, and 50 μM dithiothreitol [DTT]). The immunocomplex was incubated with GST-IκBα (catalog no. ab59981; Abcam) as the substrates in kinase buffer with 1 mM ATP-γ-S (catalog no. ab138911; Abcam) at 37°C for 30 min. Reactions were followed by addition of 2.5 mM *p*-nitrobenzyl mesylate (catalog no. ab138910; Abcam) to start alkylation for 2 h at room temperature. The reaction was stopped by boiling in 4× sample buffer and subjected to Western blotting. Membranes were probed with anti-thiophosphate ester antibody and horseradish peroxidase-conjugated secondary antibody. Labeled proteins were visualized using a LuminoGraph I.

### Statistical analyses.

Group comparisons were performed using Student’s *t* test. Data are expressed as the mean ± standard error of the mean. *P* values of <0.05 were considered statistically significant.
