# Supplementary figures and images for: Ubiquitination of SARS-CoV-2 NSP6 and ORF7a Facilitates NF-κB Activation
Source: mBio. 2022 Jul 20;13(4):e00971-22. doi: 10.1128/mbio.00971-22 (PMC9426613; doi:10.1128/mbio.00971-22)

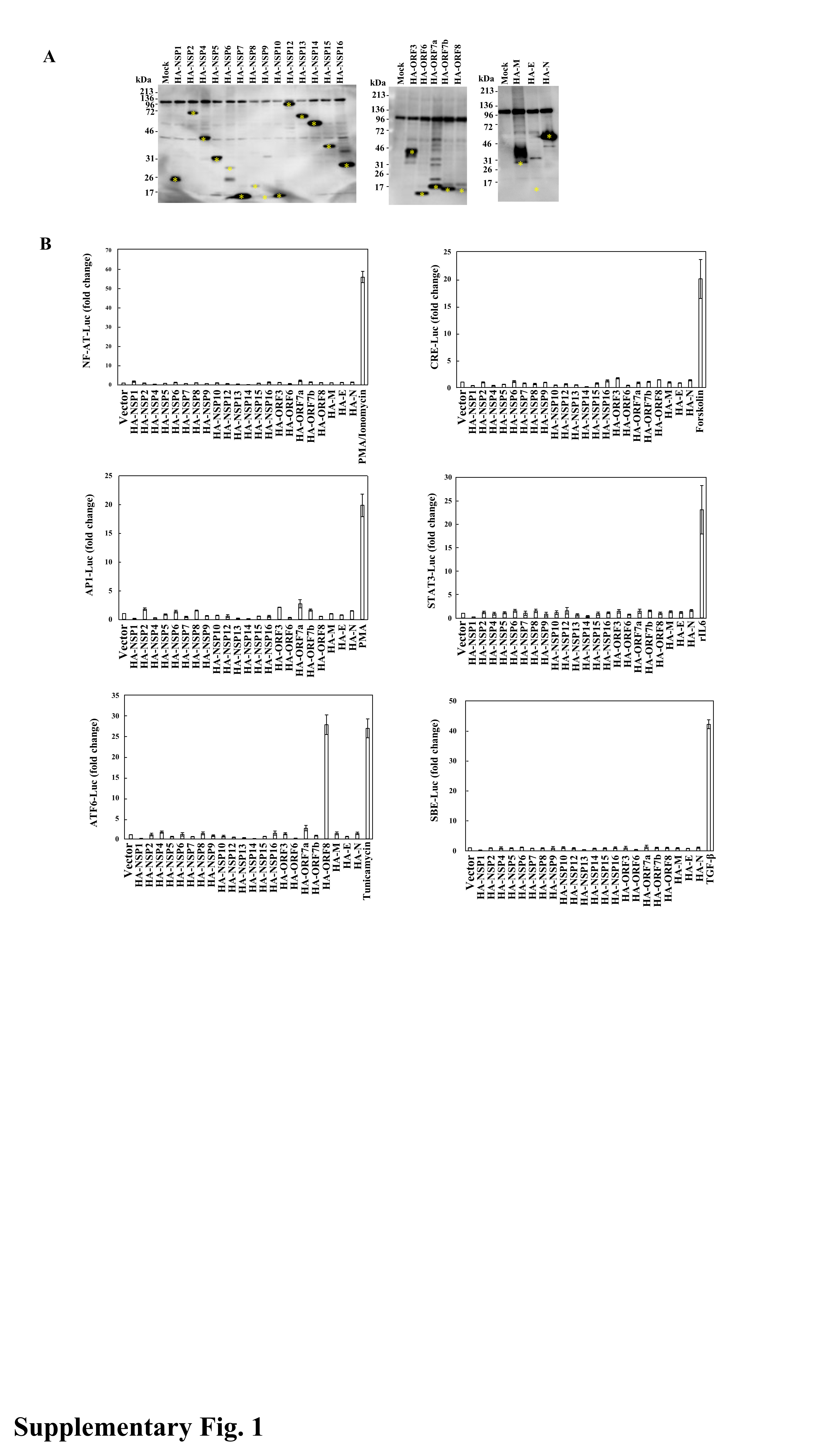

Supplement: FIG S1 [file mbio.00971-22-s0001.tif]

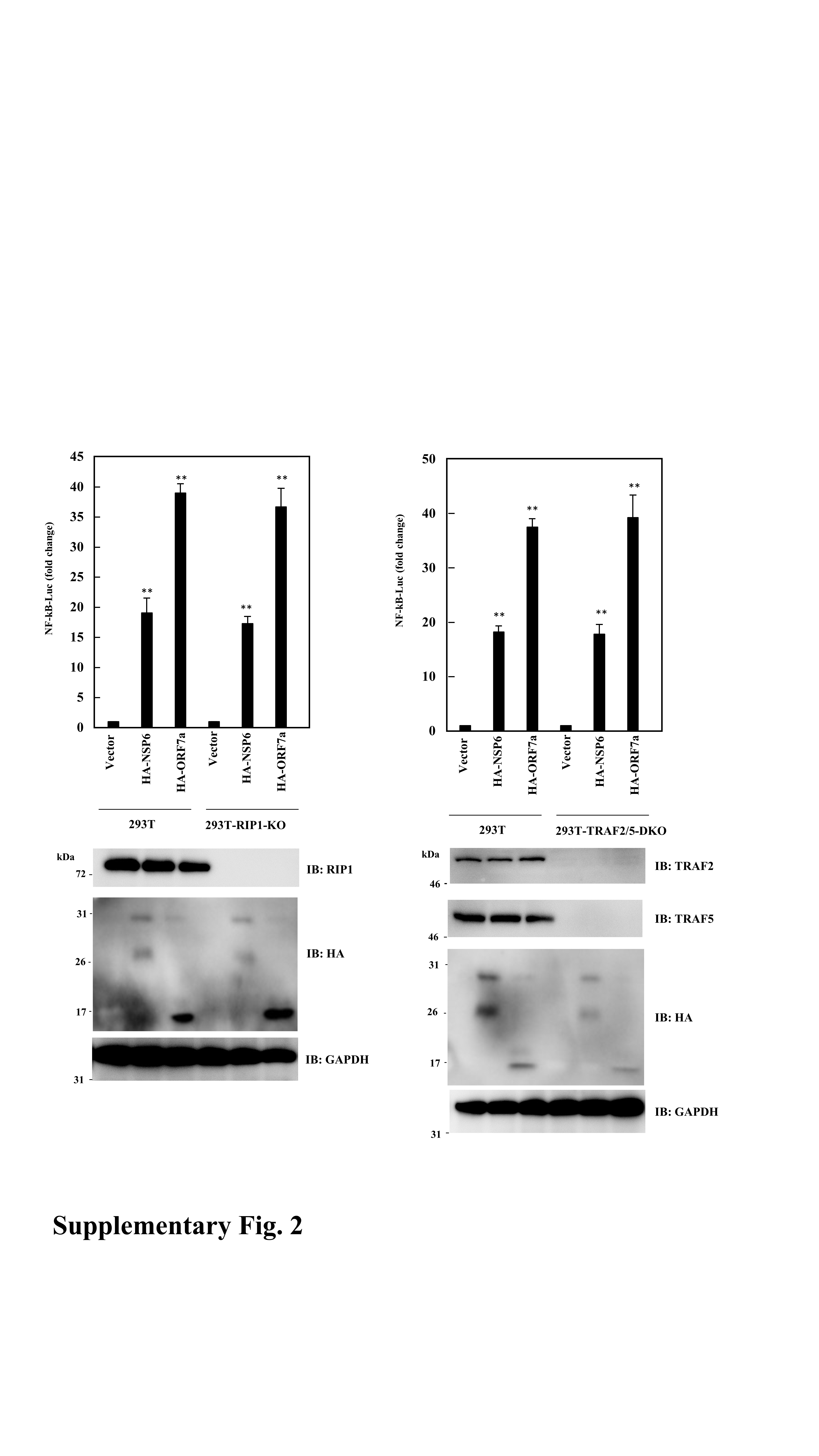

Supplement: FIG S2 [file mbio.00971-22-s0002.tif]

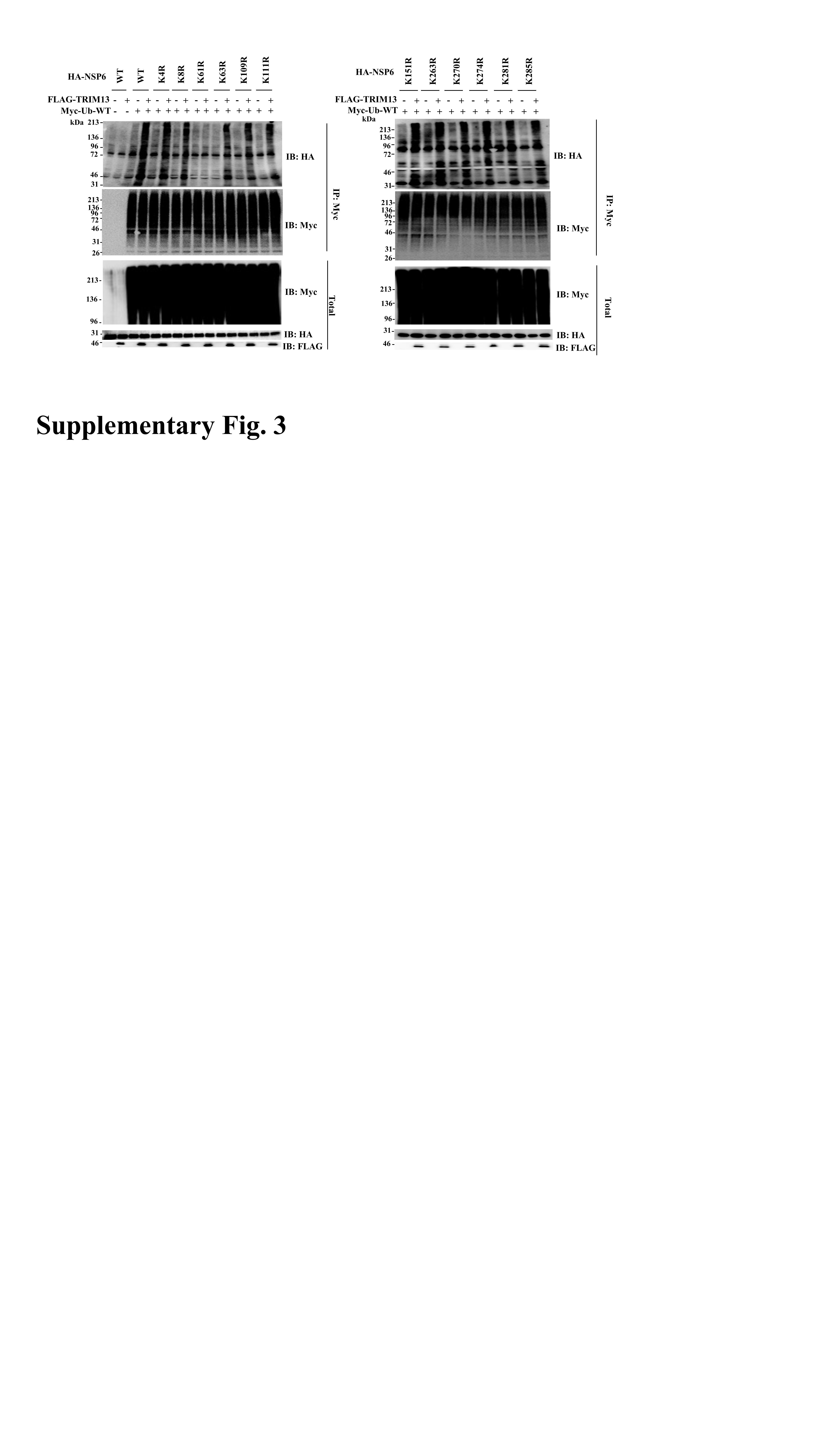

Supplement: FIG S3 [file mbio.00971-22-s0003.tif]

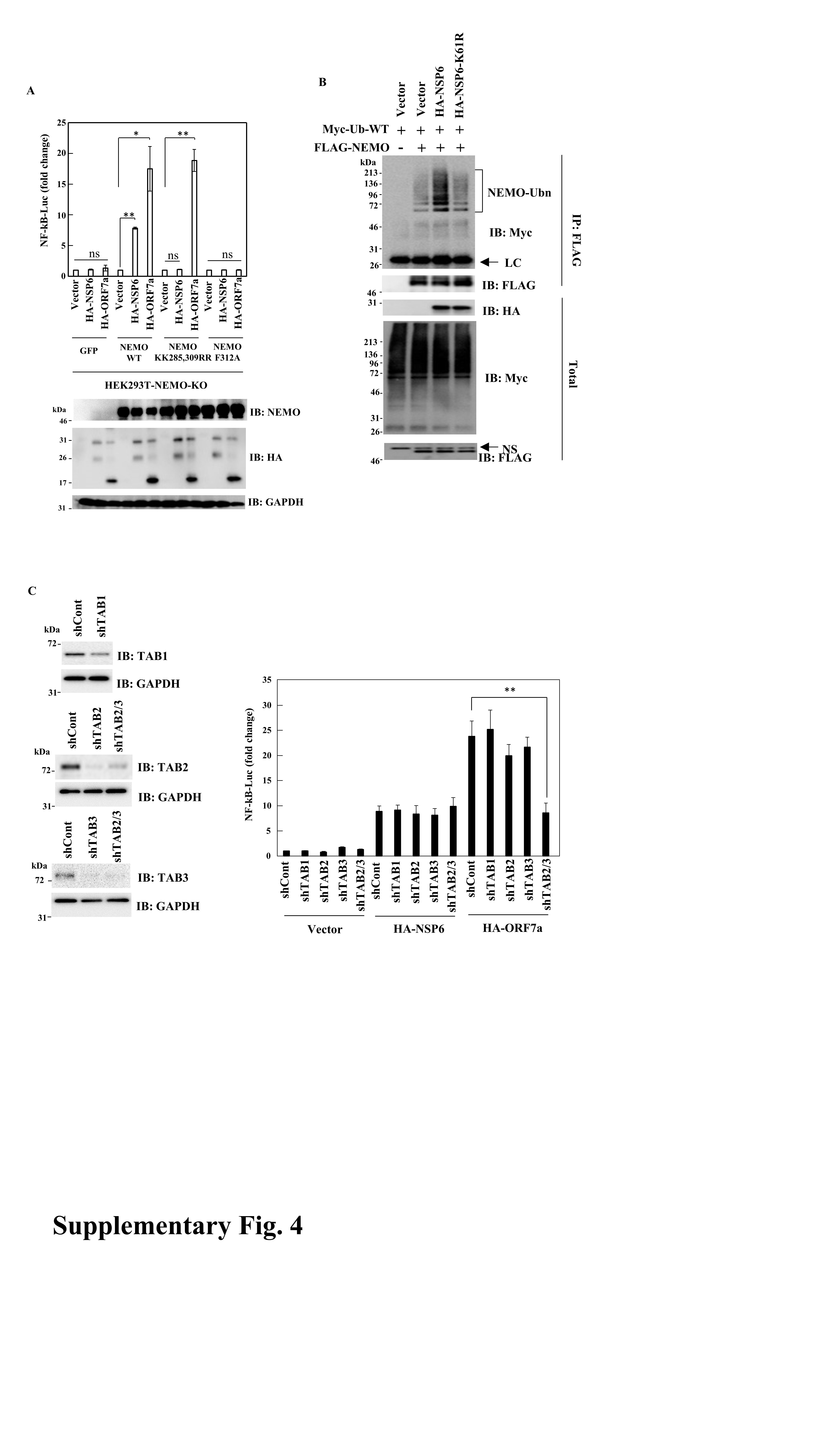

Supplement: FIG S4 [file mbio.00971-22-s0004.tif]

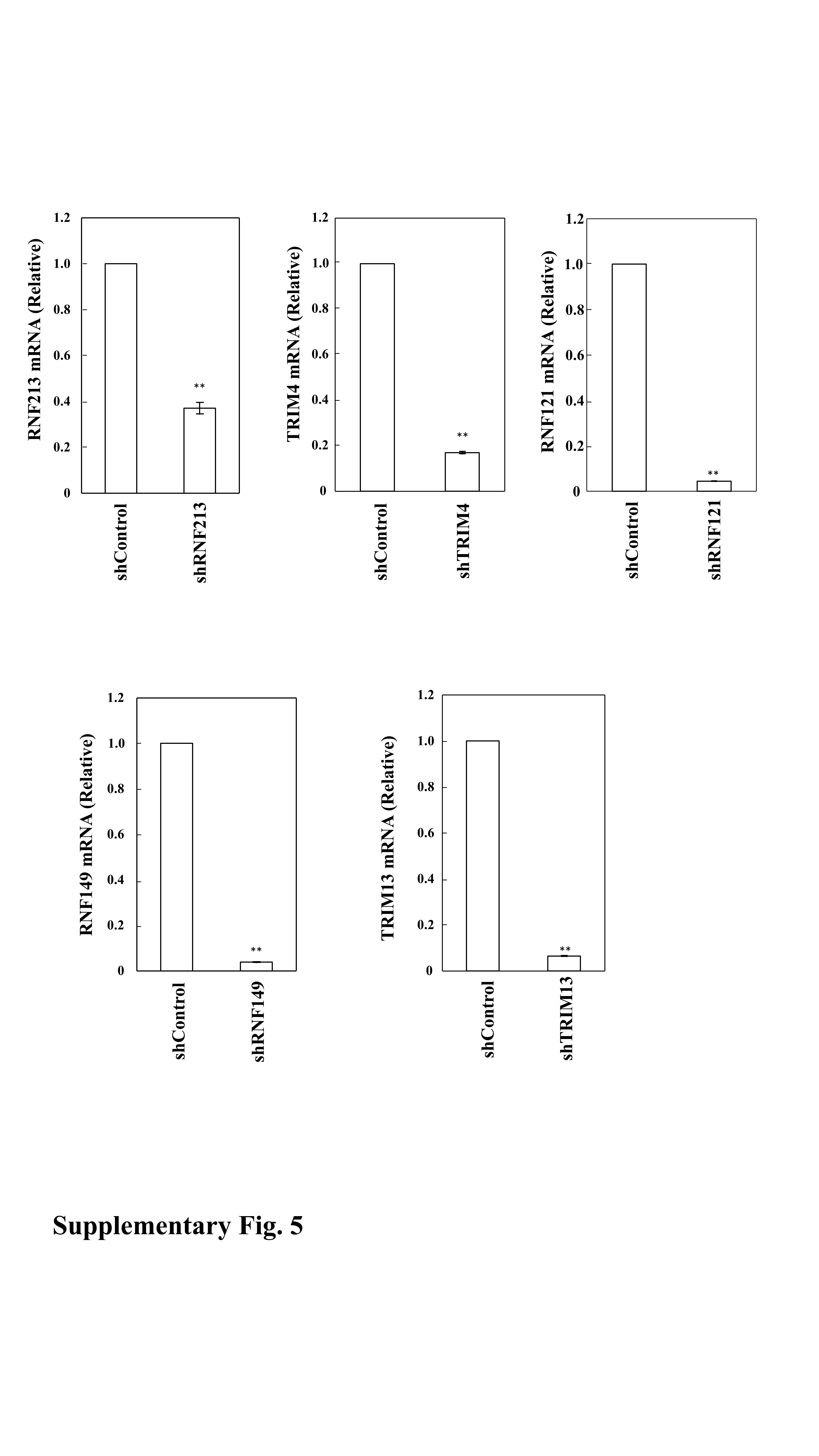

Supplement: FIG S5 [file mbio.00971-22-s0005.tif]

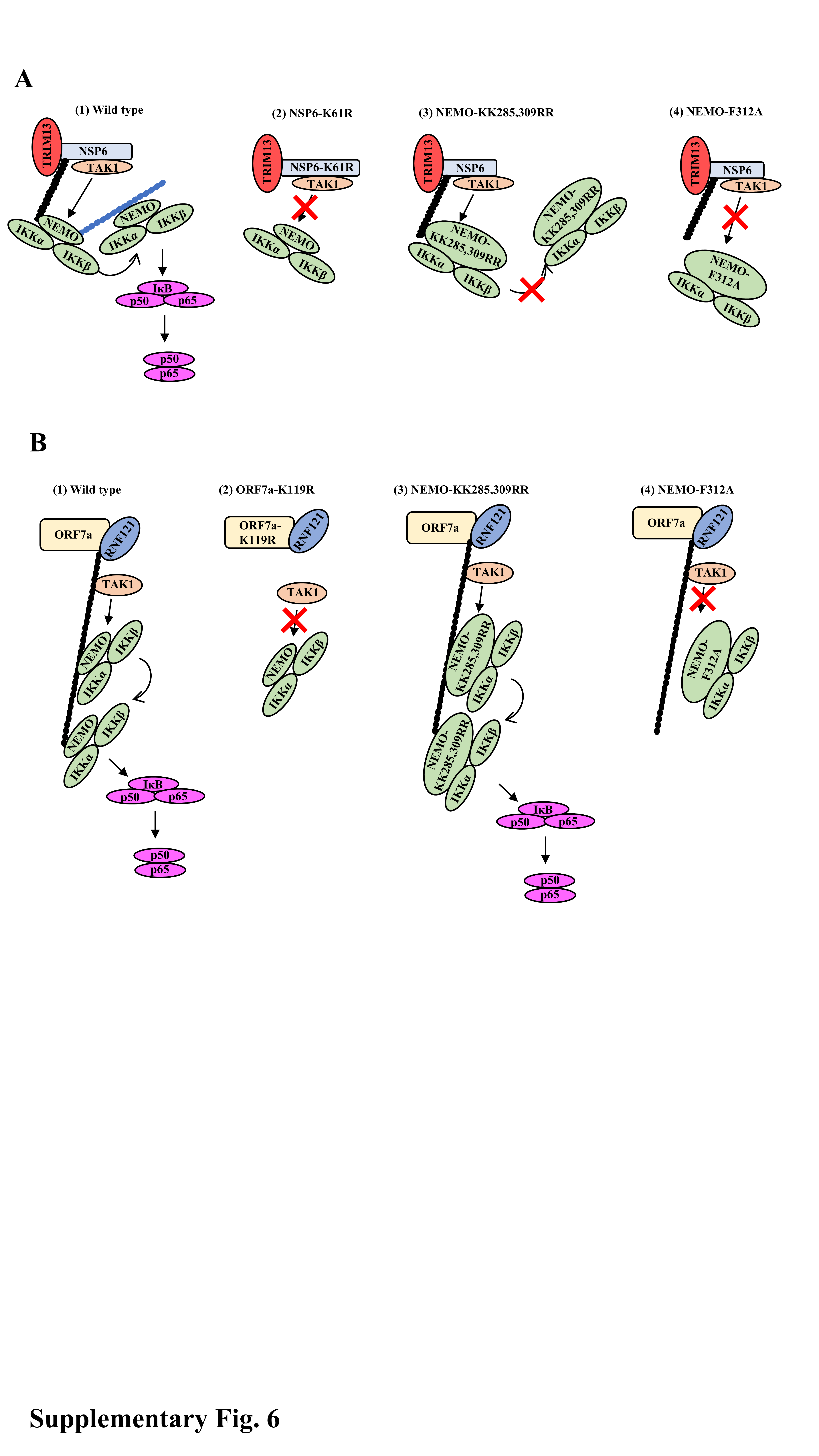

Supplement: FIG S6 [file mbio.00971-22-s0006.tif]
